# Molecular profiling of rheumatoid arthritis patients reveals an association between innate and adaptive cell populations and response to anti-tumor necrosis factor

**DOI:** 10.1186/s13075-019-1999-3

**Published:** 2019-10-23

**Authors:** Victor Farutin, Thomas Prod’homme, Kevin McConnell, Nathaniel Washburn, Patrick Halvey, Carol J. Etzel, Jamey Guess, Jay Duffner, Kristen Getchell, Robin Meccariello, Bryan Gutierrez, Christopher Honan, Ganlin Zhao, Nicholas A. Cilfone, Nur Sibel Gunay, Jan L. Hillson, David S. DeLuca, Katherine C. Saunders, Dimitrios A. Pappas, Jeffrey D. Greenberg, Joel M. Kremer, Anthony M. Manning, Leona E. Ling, Ishan Capila

**Affiliations:** 1 Momenta Pharmaceuticals Inc., 301 Binney Street, Cambridge, MA 02142 USA; 2 Corrona LLC, Waltham, MA USA; 3 DeLuca Data Science, Wietze, Germany; 4 Department of Medicine, Division of Rheumatology, Columbia University School of Medicine, New York, NY USA; 5 New York University School of Medicine, New York, NY USA; 6 Albany Medical College, Albany, NY USA

**Keywords:** Rheumatoid arthritis, TNF inhibitors, Treatment response, Innate immune system, Adaptive immune system, RNA-seq, Whole blood, Gene expression

## Abstract

**Background:**

The goal of this study is to use comprehensive molecular profiling to characterize clinical response to anti-TNF therapy in a real-world setting and identify reproducible markers differentiating good responders and non-responders in rheumatoid arthritis (RA).

**Methods:**

Whole-blood mRNA, plasma proteins, and glycopeptides were measured in two cohorts of biologic-naïve RA patients (*n* = 40 and *n* = 36) from the Corrona CERTAIN (Comparative Effectiveness Registry to study Therapies for Arthritis and Inflammatory coNditions) registry at baseline and after 3 months of anti-TNF treatment. Response to treatment was categorized by EULAR criteria. A cell type-specific data analysis was conducted to evaluate the involvement of the most common immune cell sub-populations. Findings concordant between the two cohorts were further assessed for reproducibility using selected NCBI-GEO datasets and clinical laboratory measurements available in the CERTAIN database.

**Results:**

A treatment-related signature suggesting a reduction in neutrophils, independent of the status of response, was indicated by a high level of correlation (*ρ* = 0.62; *p* < 0.01) between the two cohorts. A baseline, response signature of increased innate cell types in responders compared to increased adaptive cell types in non-responders was identified in both cohorts. This result was further assessed by applying the cell type-specific analysis to five other publicly available RA datasets. Evaluation of the neutrophil-to-lymphocyte ratio at baseline in the remaining patients (*n* = 1962) from the CERTAIN database confirmed the observation (odds ratio of good/moderate response = 1.20 [95% CI = 1.03–1.41, *p* = 0.02]).

**Conclusion:**

Differences in innate/adaptive immune cell type composition at baseline may be a major contributor to response to anti-TNF treatment within the first 3 months of therapy.

## Background

Disease-modifying anti-rheumatic drugs (DMARDs) are the cornerstone of anti-inflammatory therapy in rheumatoid arthritis (RA), with patients who do not respond to traditional synthetic DMARDs usually initiating therapy with TNF inhibitors [1, 2]. TNF inhibitors may take several months to exert their effect, and for those, approximately 30–40% of RA patients, who do not respond adequately to the anti-TNF therapy, an alternative agent is chosen. It is not uncommon for RA patients to undergo therapy with multiple biologics before an agent or combination of agents that induce remission is eventually found. The resulting delay in controlling disease activity may result in joint damage disability and increased the cost of treatment. For these reasons, research is being devoted to identifying factors influencing response to anti-TNF therapy in RA.

The present understanding of the associations between various factors (e.g., demographic, clinical, and genetic) and the probability of a given RA patient to respond to anti-TNF therapy has been extensively reviewed [3, 4]. Although some of these markers have been proposed for informing the choice of biologic treatment in RA [5], identification of baseline predictors for patient response to anti-TNF therapy in RA that reproducibly manifest clinically relevant predictive value remains an unsolved problem [6, 7].

Replication of the findings of potential clinical utility for candidate biomarkers has been particularly challenging due to heterogeneity of RA patient population (and consequently, variability in patients’ cohorts evaluated by different studies), the expected multiplicity of factors influencing patients’ response to therapy (e.g., genetic, immunologic, and environmental), differences in outcomes used and definitions of what constitutes response to therapy across the studies, and the diversity of analytical approaches characterizing patient samples.

Heterogeneity of inflammatory, immunologic, and tissue remodeling phenotypes of RA patients has been revealed by gene expression profiling of synovial samples [8, 9]. Such diversity of the RA patient population has been found to influence their response to anti-TNF therapy [10] with increased infiltration of TNF-secreting cells in good responders [9]. Replication of these results in multiple independent studies has likely been hindered by limited availability of synovial biopsies as well as compositional variability of these samples [11].

Molecular characterization of peripheral blood samples is particularly appealing given the relative ease of obtaining samples as part of patient follow-up. Gene expression profiling of whole blood before and after anti-TNF treatment showed significant changes in multiple co-expression gene modules that have been replicated in several patient cohorts [12]. A recent study has also evaluated the impact of treatment on molecular measures (gene expression, proteomics, and cell counts) in RA patients when compared to a healthy control group [13]. Lower consistency has been observed across the findings from the studies [14–17] investigating the association between differences in molecular profiles of blood samples from RA patients at baseline and their response to anti-TNF therapy [18]. Some of the differences among their results might be attributed to the diversity of the gene arrays [14, 15] and definitions of response to the treatment [14, 16, 17]. Findings from more recent investigations of the relationship between baseline gene expression profiles of blood samples from RA patients and their response to treatment with TNF inhibitors are also mixed, with one study reporting associations between non-response and increased plasma and B cell markers [19], whereas another finds a lack of consistent differences between co-expression patterns for good and poor responders across multiple patient cohorts [12].

The current study is a comprehensive molecular profiling of plasma and whole-blood RNA samples collected from two cohorts of biologic-naïve RA patients from the Corrona CERTAIN registry [20] immediately prior to initiation of anti-TNF treatment (baseline; BL) and following 3 months of therapy (MO3). Resulting measurements enabled characterization of changes in molecular profiles of RA patients following anti-TNF treatment and the associations between baseline patient characteristics and their response to anti-TNF therapy in a real-world setting. Particular emphasis was made on evaluating reproducibility of these findings between these two cohorts as well as in relevant publicly available data. Additionally, observations related to the relationship between innate/adaptive cell type composition of baseline samples and patient response to anti-TNF treatment were confirmed for a larger group of CERTAIN RA patients using available complete blood count (CBC) lab measurements.

## Methods

### Study design and sample selection criteria

RA patient characteristics and samples were obtained from the Corrona CERTAIN study (NCT01625650) [20]. CERTAIN is a prospective, nonrandomized, comparative effectiveness cohort study nested within the US Corrona registry and includes adult patients with RA who have at least moderate disease activity (CDAI > 10) and are starting or switching biologics. Within CERTAIN, biologic samples were collected at baseline (start of biologic) and at 3- and 6-month follow-up visits. Patients were followed up for up to 12 months on drug.

This investigation included RA patients from CERTAIN that were biologic naïve (i.e., no prior treatment with a biologic agent), initiating treatment with adalimumab or infliximab in conjunction with methotrexate (MTX) and no or stable low-dose prednisone (< 5 mg). Two cohorts of good responders (EULAR-GR) and non-responders (EULAR-NR) according to EULAR criteria on DAS28-CRP [21, 22] for clinical response to therapy at 3 months were selected for RNA-seq, proteomics, and targeted glycopeptide analysis. Patient consent and IRB approval were obtained as described in [20]. EULAR-GR and EULAR-NR were chosen to represent more pronounced good and non-response and approximately balanced within each cohort with respect to selected criteria at baseline (e.g., age, education, smoking, BMI, disease duration, CRP, and SJC28). All patients have been on MTX treatment and had at least moderate disease activity at their entry in the study [20] with DAS28-CRP for the majority (> 60%) of the patients in each cohort exceeding the high disease activity cutoff [23] indicating that they were not adequately responding to prior MTX therapy. Propensity scores based on these attributes were used to select EULAR-GR and EULAR-NR with resulting scores in the area of common support. Non-responders with adalimumab or infliximab levels below 800 ng/mL in MO3 plasma samples were excluded (Additional file 1: Supplementary methods). The final two cohorts included 40 [cohort 1 (C1): 19 EULAR-GR and 21 EULAR-NR] and 36 [cohort 2 (C2): 21 EULAR-GR and 15 EULAR-NR] RA patients. C2 samples were selected and analyzed independently from C1 samples 18–24 months later. Samples from each cohort have been randomized prior to sample processing.

### Proteomics analysis by LC-MS/MS

Plasma samples depleted of the most abundant proteins were trypsin/Lys-C digested and separated by HPLC prior to mass spectrometry. Searches against human Uniprot were performed by Sequest HT in ProteomeDiscoverer 1.4. Further details are provided in Additional file 1: Supplementary methods.

### FcγRIIIb genotyping by glycopeptide analysis

Allelic variants of FcγRIIIb single nucleotide polymorphisms (SNPs) have been assigned through the quantitation of corresponding peptide and glycopeptide markers. Targeted nLC-MS/MS for assignment of allelic variants was conducted as described previously [24].

### RNA preparation and NGS sequencing (RNA-seq)

RNA extracted from whole-blood PAXgene tubes (Qiagen) was poly-A enriched prior to library construction according to the manufacturers’ protocols. Sequencing was performed on Illumina HiSeq 2500 system. FASTQ files were mapped to human reference (UCSC hg19) genome using two-pass STAR alignment [25]. QC metrics of resulting BAM files were obtained using RNA-SeQC [26]. Resulting gene-level fragment counts generated by featureCounts [27] were deposited to NCBI-GEO (GSE129705). Additional details are provided in Additional file 1: Supplementary methods.

### Data analysis

Descriptive statistics were used to summarize baseline characteristics for the EULAR-GR and EULAR-NR in both cohorts. Two sample Wilcoxon tests (continuous variables) and chi-square tests of association (categorical variables) were used to compare baseline characteristics between EULAR-GR and EULAR-NR within each cohort. Differential gene and protein expression analyses used limma-voom methodology [28, 29]. Benjamini-Hochberg false discovery rate (BH-FDR) [30] was used for multiple test correction. Global concordance of differential expression was assessed by rank correlation and permutation controls at 5% significance level. Adjustment for confounding factors (e.g., subject variability and sample processing order) was accomplished by including them into statistical models. Further details are provided in Additional file 1: Supplementary methods. Logistic regression was used to evaluate the association between baseline CBC metrics and EULAR response without covariate adjustment and adjusted by a priori selected variables (drug group, age, smoking status, disease duration, modified HAQ, concomitant MTX use, and number of prior biologics—all at the time of initiation of anti-TNF therapy).

## Results

### Characteristics of the patients

Summary of baseline demographic and clinical attributes for EULAR-GR and EULAR-NR for each cohort is presented in Table 1 (see Additional file 1: Table S1 for subjects with gene expression data available at baseline). The majority of the attributes are comparable between EULAR-GR and EULAR-NR and between the two cohorts. Disease duration is higher for C2 samples with differences between EULAR-GR and EULAR-NR trending in the opposite direction between two cohorts [higher in EULAR-GR in C1 (*p* = 0.023), marginally elevated in EULAR-NR in C2 (*p* = 0.5)]. Higher percentages of CCP and RF-positive subjects are observed for EULAR-GR in both cohorts (*p* < 0.05 for CCP in both cohorts) compared to EULAR-NR. DAS28-CRP and TJC28 are higher for EULAR-NR in each cohort (*p* < 0.05 for C1 and pooled data from both cohorts) compared to EULAR-GR. For the remaining attributes, none of the differences between EULAR-GR and EULAR-NR in either cohort were statistically significant at *p* = 0.05 level.

**Table 1:** Demographic and clinical characteristics of cohorts 1 and 2. GR and NR indicate EULAR good responders and non-responders respectively. Numbers in brackets after each attribute represent percentages or standard deviation (SD) of that attribute, as indicated

	Cohort 1			Cohort 2		
	GR	NR	*p*	GR	NR	*p*
*N*	19	21	N/A	21	15	N/A
Female, *N* (%)	15 (79)	19 (90)	0.56	16 (76)	12 (80)	1
Age^§^, mean (SD)	54 (13)	56 (13)	0.48	55 (12)	51 (9.9)	0.41
White, *N* (%)	17 (89)	14 (67)	0.18	19 (90)	13 (87)	1
BMI^§^, mean (SD)	29 (7.6)	30 (6.3)	0.45	30 (7)	33 (7.1)	0.092
College educated^§^, *N* (%)	10 (53)	13 (62)	0.79	12 (57)	10 (67)	0.82
Non-smoker^§^, *N* (%)	8 (42)	14 (67)	0.21	14 (67)	9 (60)	0.95
Current or previous smoker^§^, *N* (%)	11 (58)	7 (33)	0.21	7 (33)	6 (40)	0.95
Infliximab, *N* (%)	8 (42)	9 (43)	1	6 (29)	8 (53)	0.25
Adalimumab, *N* (%)	11 (58)	12 (57)	1	15 (71)	7 (47)	0.25
SJC28 [BL]^§^, mean (SD)	6.7 (3.7)	9.1 (5.5)	0.18	9.6 (5.5)	8.7 (4.9)	0.75
TJC28 [BL], mean (SD)*	9 (6.2)	15 (8.3)	0.027	11 (6.7)	14 (5.7)	0.16
ln (CRP) [BL]^§^, mean (SD)	1.6 (1.6)	1.2 (1.8)	0.53	1.5 (1.4)	1.8 (1.1)	0.43
DAS28-CRP [BL], mean (SD)*	4.5 (0.78)	5.2 (0.94)	0.016	4.8 (0.83)	5.2 (0.66)	0.054
RA duration^§^, mean (SD)*	5.4 (7.5)	1.9 (1.7)	0.023	5 (6.5)	7.2 (8.3)	0.5
RF+, *N* (%)	16 (84)	12 (57)	0.13	16 (76)	8 (53)	0.28
CCP+, *N* (%)*	16 (84)	8 (38)	0.0081	17 (81)	6 (40)	0.03

*Difference between good and non-responders at baseline for this attribute is statistically significant (*p* < 0.05) in at least one of the cohorts

^§^Propensity score informing selection of the patients was based on age, level of education, smoking history, BMI, duration of disease, and baseline CRP and SJC28

### Molecular signature of anti-TNF treatment

**Fig. 1:**
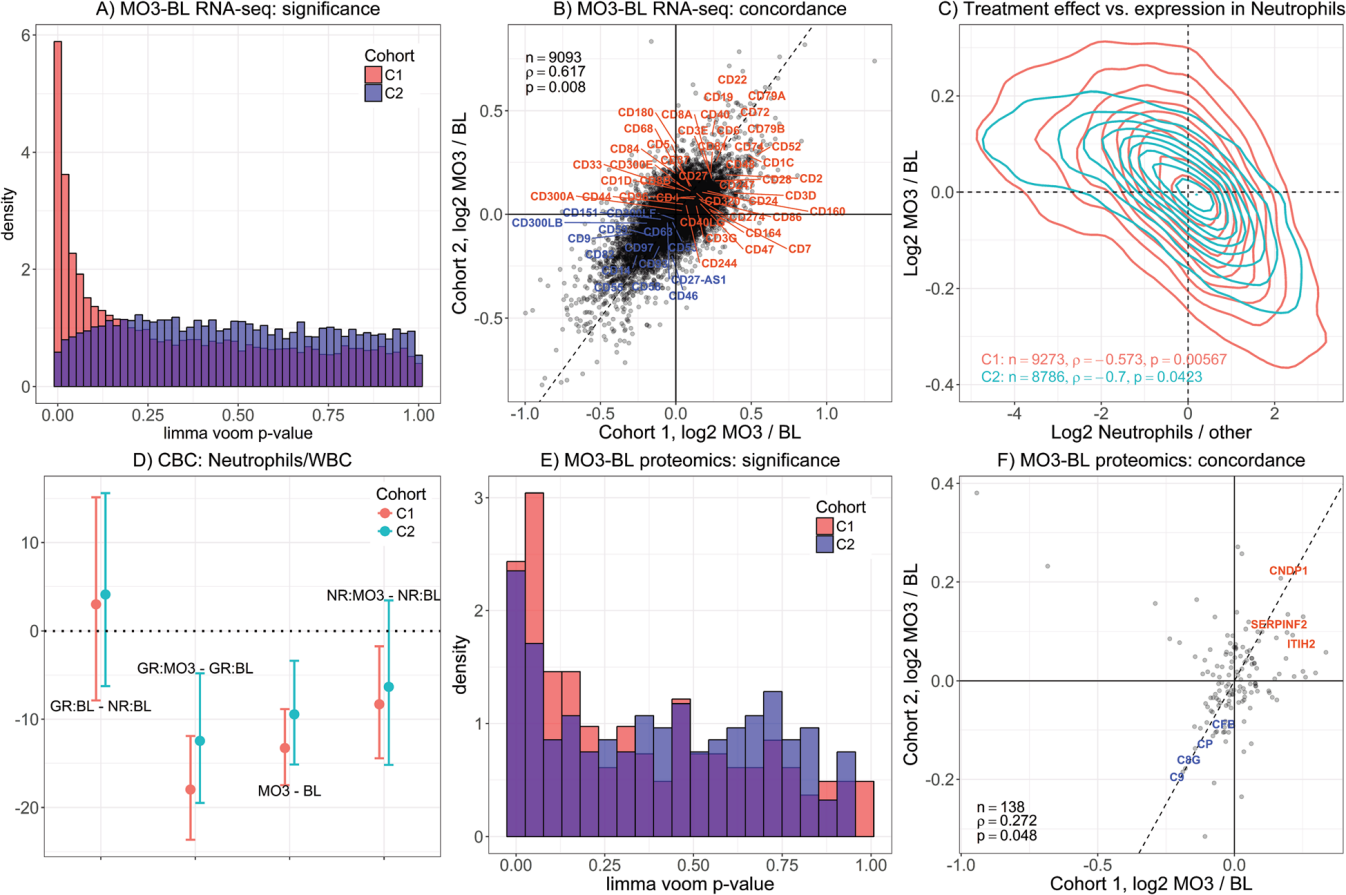
Pharmacodynamic effects of anti-TNF treatment in gene expression, proteomics, and CBC data. **a** Gene expression profiles show statistically significant differences (after FDR correction) between the month 3 and baseline samples in C1 (pink), but not in C2 (blue). **b** Scatterplot of mean MO3-BL differences in gene expression levels for cohort 1 (*x*-axis) and cohort 2 (*y*-axis). Color labels indicate CD cell surface markers up- (orange) or downregulated (blue) in both cohorts. **c** 2D density contours of genome-wide mean MO3-BL gene expression differences (*y*-axis) and log-fold differences in expression levels between neutrophils and the rest of cell types in NCBI-GEO dataset GSE60424 (*x*-axis): genes overexpressed in neutrophils are downregulated at 3 months in both cohorts. **d** Average differences (%) and 95% confidence intervals on the neutrophils/WBC ratios in CBC metrics for EULAR-GR and EULAR-NR at BL and MO3. **e** Distribution of treatment (MO3-BL) effect *p* values in plasma proteomics analysis manifests an increase in small *p* values for both cohorts. **f** Average MO3-BL differences in plasma protein levels show a positive correlation between two cohorts infrequently observed upon permutation. Labels indicate proteins with BH-FDR < 20% in both cohorts

Genome-wide differences between MO3 and BL gene expression levels were evaluated across patients in each cohort, irrespective of their EULAR response status. Distribution of *p* values (Fig. 1a) shows substantial numbers of genes achieving low BH-FDR levels (775 genes at BH-FDR < 0.05) in C1, but not in C2 (3 genes at BH-FDR < 0.05). The treatment effect manifests a strong positive correlation of the mean MO3-BL differences between the two cohorts (Fig. 1b). The majority of genes exhibiting the largest MO3-BL differences in both cohorts are downregulated and related to myeloid cells and platelets (Additional file 2: Table S3 and S4). Granulocyte biology appears to be modulated, including functions related to degranulation, chemotaxis, and migration. The majority of the upregulated genes are involved in protein synthesis, including transcription, translation, and ribosome-related genes (Additional file 2: Table S3 and S4). The most significantly modulated cell surface markers (Fig. 1b) include T and B cell marker (i.e., CD3, CD4, CD8, CD79, CD22, and CD52) that are upregulated in both cohorts, while myeloid markers (CD14, CD55, CD46) are downregulated. The modulation of cell types by treatment was assessed using cell type-specific RNA-seq dataset as reference [31]. In both cohorts, neutrophil-related genes show the most significant negative correlation with the effect of treatment (Fig. 1c, Additional file 2: Table S5), while, conversely, B cell and CD4/CD8 T cell-specific genes were positively correlated (Additional file 1: Figure S1). These results were robust to the choice of cell type-specific reference dataset (Additional file 1: Figure S2). Finally, analysis of CBC data showed that, on average, the neutrophil/white blood cell (WBC) ratio at MO3 is 87% of that at baseline (95% CI = [83%, 91%]; *p* = 1.2 × 10^−6^) for C1 and 91% (95% CI = [85%, 97%]; *p* = 0.004) for C2 (Fig. 1d).

Analysis of differential protein expression in plasma by shotgun proteomics was limited to the most reliably quantified proteins (C1: 159 and C2: 181). Statistically significant MO3-BL differences have been detected in both cohorts (C1: 14, C2: 9 proteins at BH-FDR < 0.05 in each cohort, permutation *p* < 0.001 in both cohorts) (Fig. 1e). The average differences of protein expression levels show positive correlation between the two cohorts across all proteins included in the analysis, which was infrequently observed upon permutation (*ρ* = 0.27, *p* = 0.05) (Fig. 1f). Gene Ontology (GO) analysis revealed a downregulation of inflammatory pathways, without discriminating between innate and adaptive immune processes (Additional file 2: Table S6). Conversely, proteins mostly synthesized in the liver, including fibronectin, plasminogen, apolipoprotein E, and proteins that are not involved in immune functions (i.e., SERPINF1/PEDF, HSPA5/BiP) are increased. Inclusion of less abundant proteins in the analysis showed a decrease of more than 30% in each cohort (*p* ≤ 0.01) for the acute phase proteins haptoglobin and C-reactive protein (CRP) upon treatment.

### Association between anti-TNF treatment signature and clinical response

**Fig. 2:**
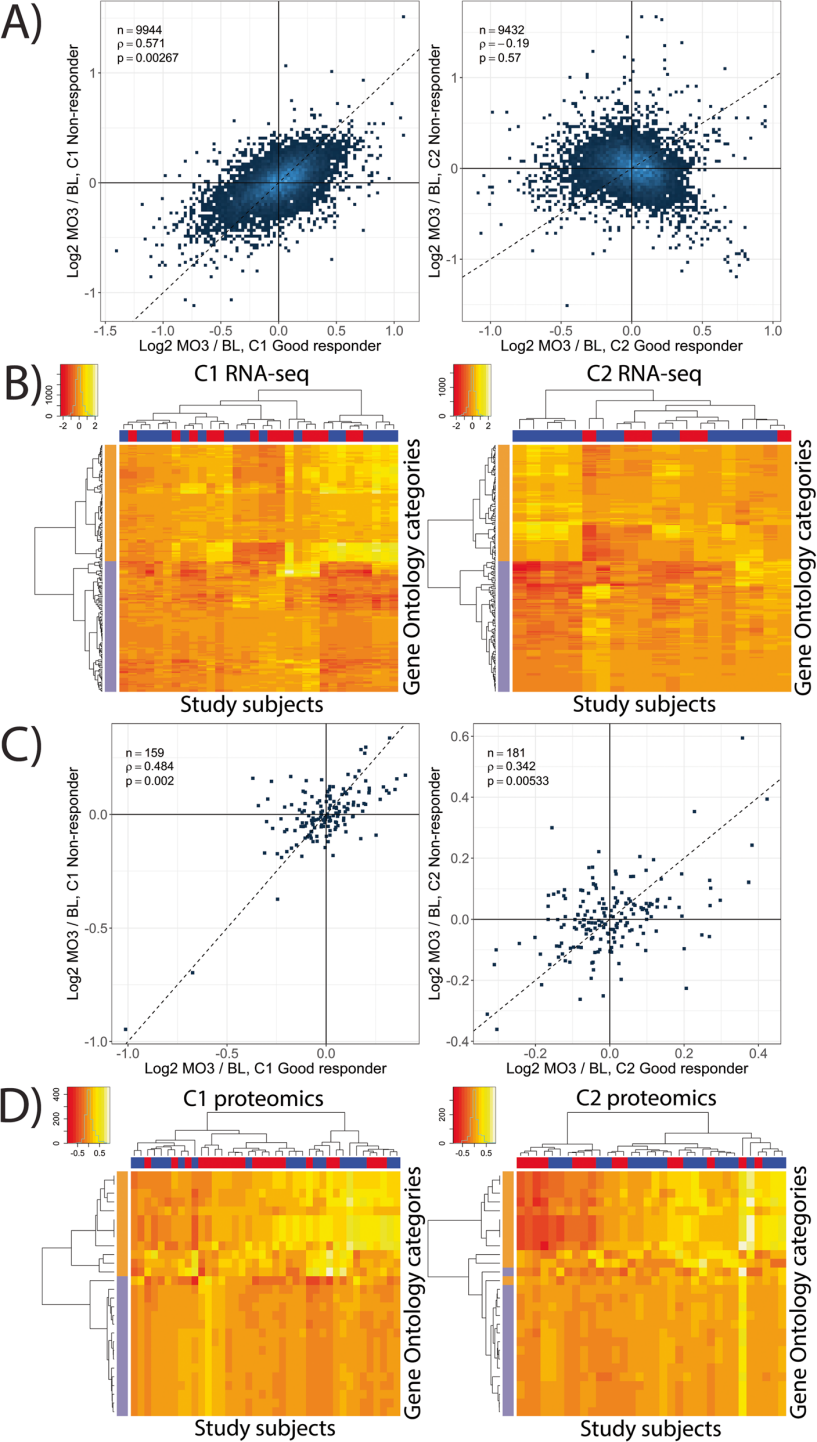
Effect of anti-TNF treatment in EULAR-GR and EULAR-NR in C1 and C2. **a** At an individual gene level, the gene expression profile changes upon anti-TNF treatment are highly correlated between EULAR-GR and EULAR-NR, except for EULAR-NR from C2. **b** MO3-BL differences in gene expression averaged for GO categories most up/downregulated upon treatment (BH-FDR < 0.01) cluster by the direction of the treatment (orange—up, purple—downregulated at MO3), but not by clinical response (EULAR-GR—blue, EULAR-NR—red) in both cohorts. **c** Statistically significant positive correlation of MO3-BL differences in protein levels is observed between EULAR-GR and EULAR-NR in each cohort. **d** MO3-BL differences in plasma protein levels averaged for GO categories most up/downregulated upon treatment (BH-FDR = 0.2) cluster predominantly by the direction of the treatment (orange—up, purple—downregulated at MO3), but not by clinical response (EULAR-GR—blue, EULAR-NR—red) in both cohorts

To compare molecular signatures of anti-TNF treatment in EULAR-GR and EULAR-NR, MO3-BL differences in gene expression levels have been estimated separately for EULAR-GR and EULAR-NR in each cohort. The significance of correlations between MO3-BL differences for each set of subjects has been estimated by permutation. Except for EULAR-NR from C2, the remaining three groups of subjects display significant pairwise correlations of MO3-BL differences (Fig. 2a and Additional file 1: Figure S3). The differences between the treatment effects in EULAR-GR and EULAR-NR did not achieve statistical significance upon permutation neither for individual genes nor for Gene Ontology categories in C1 or C2 (BH-FDR > 0.5). This suggests that the anti-TNF treatment effect on gene expression is similar in both EULAR-GR and EULAR-NR. This observation is also concordant with publicly available data (Additional file 1: Table S7 and Additional file 1: Figure S4). The lack of discrimination between EULAR-GR and EULAR-NR was further confirmed following GO analysis of the genes affected by anti-TNF treatment (Fig. 2b).

Similar to gene expression, changes in proteomics data following anti-TNF treatment are positively correlated (*ρ* = 0.48, *p* = 0.002 for C1; *ρ* = 0.34, *p* = 0.005 for C2) between EULAR-GR and EULAR-NR in both cohorts (Fig. 2c). Good and non-responders also cannot be discriminated based on modulated pathways (Fig. 2d). Changes in cell populations by CBC, however, show a greater decrease in the neutrophils/WBC ratio from BL to MO3 in EULAR-GR than in EULAR-NR in both cohorts (by 10.5% and 6.5% in C1 and C2, respectively), achieving statistical significance only for C1 (95% CI = [− 19%, − 1.6%]; *p* = 0.03), but not for C2 (95% CI = [− 18%, 5.9%]; *p* = 0.30). Overall, those results indicate that the molecular signature of anti-TNF treatment is independent of the status of response. Additional factors must be likely contributing to the development of demonstrable clinical response to anti-TNF treatment.

### Differences between good responders and non-responders at baseline

**Fig. 3:**
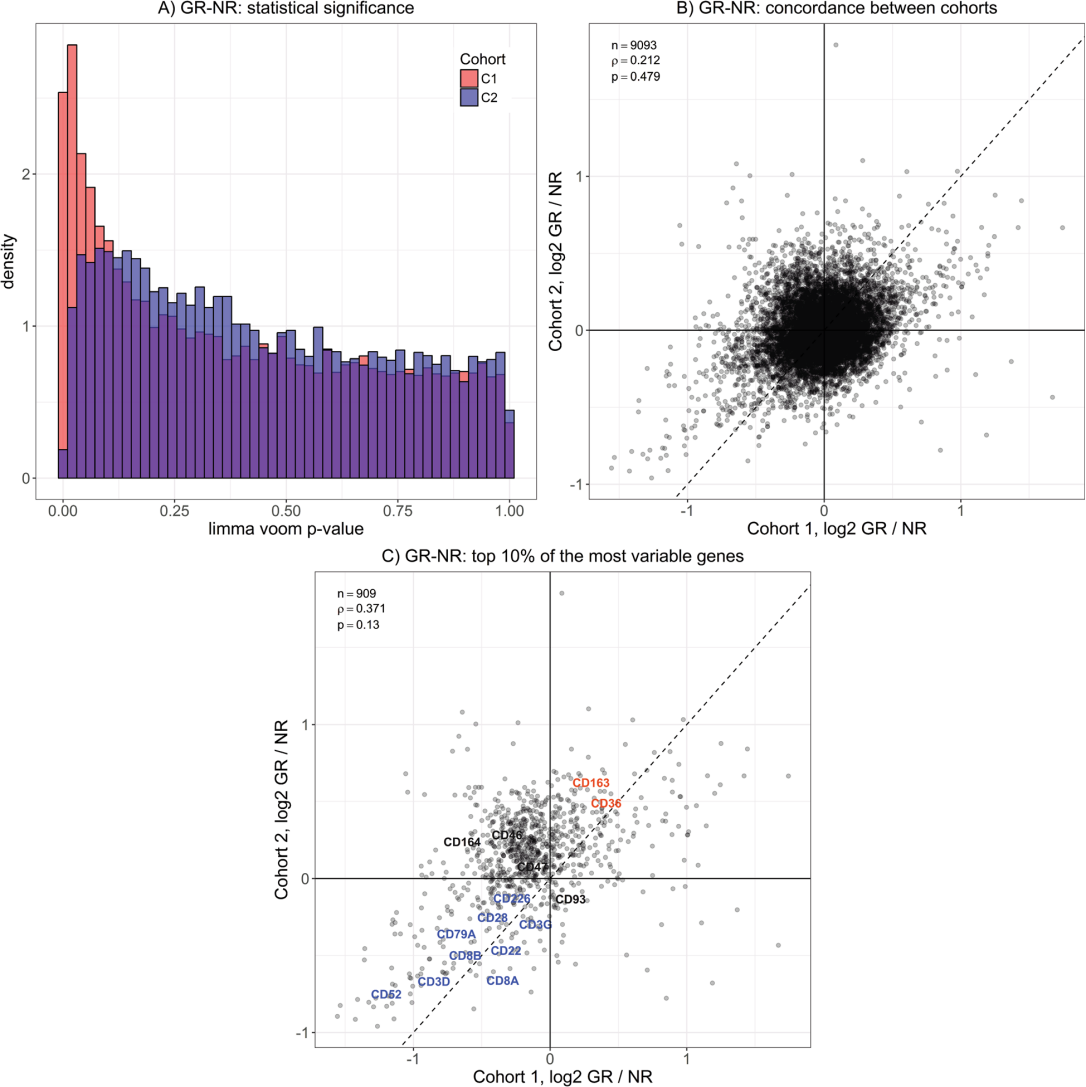
Analysis of baseline differences in gene expression between EULAR-GR and EULAR-NR. **a** Statistically significant (after BH-FDR correction) differences were observed in C1 (blue), but not in C2 (pink). **b** Genome-wide correlation of average EULAR-GR–EULAR-NR differences at baseline across both cohorts was positive, but not statistically significant by permutation control. **c** Baseline EULAR-GR–EULAR-NR differences for a subset of more variable genes show greater positive correlation between two cohorts that is less frequently observed upon permutation and includes cell surface markers for myeloid cells (CD136, CD63) higher on average in EULAR-GR in both cohorts (orange) and lymphocytes (e.g., CD52 and CD22) on average higher in EULAR-NR in both cohorts (blue)

The comparison between EULAR-GR and EULAR-NR at baseline demonstrated only modest differences. In particular, shotgun proteomics data did not yield noteworthy findings (BH-FDR > 0.5; *ρ* = 0.025; *p* = 0.85). Differences between gene expression levels achieved statistical significance in C1 (77 and 536 genes at BH-FDR cutoffs of 0.1 and 0.2 respectively) but not in C2 (lowest BH-FDR of 0.73) (Fig. 3a). Similarly, although differences between baseline gene expression levels in EULAR-GR and EULAR-NR between two cohorts are positively correlated, no statistical significance was achieved by permutation control (*ρ* = 0.21; *p* = 0.48) (Fig. 3b).

**Fig. 4:**
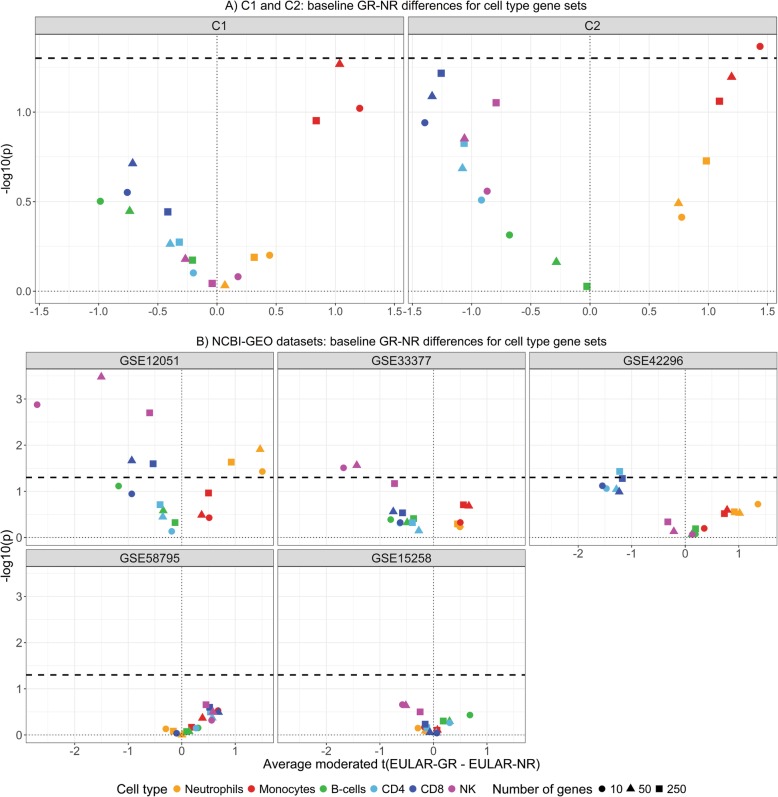
Cell type-specific gene expression analysis between EULAR-GR and EULAR-NR at baseline. **a** Volcano plots of genes predominantly expressed in specific cell types in the blood shows that across both cohorts genes specific to monocytes and neutrophils are expressed (on average) higher in EULAR-GR whereas those specific to B cell and T cells are expressed (on average) higher in EULAR-NR. The horizontal line corresponds to a permutation *p* value of 0.05. **b** Same analysis was applied to five publicly available datasets. A similar observation was made in three of the five datasets

However, differences between EULAR-GR and EULAR-NR become more highly correlated between the two cohorts when the analysis is restricted to more variable genes (Additional file 1: Figure S5). The top 10% of the most variable genes includes cell surface markers that are associated with myeloid cells (CD14, CD36, CD46, CD47, CD163, and CD164) and are higher on average in EULAR-GR, while surface markers for lymphocytes, including T cells (CD52, CD48, CD3D, CD8A) and B cells (CD79B, CD22), are on average higher in EULAR-NR in both cohorts, suggesting differences in adaptive/innate balance between EULAR-GR and EULAR-NR at baseline (Fig. 3c). In both cohorts, genes that are most expressed in innate immune cells (neutrophils and monocytes) were, on average, found to be expressed at higher levels in EULAR-GR, while genes predominantly expressed in the adaptive compartment (CD4/CD8/NK/B cells) were on average higher in EULAR-NR (Fig. 4a). The significance of this observation is supported by permutation when assessed across both cohorts and is robust to the choice of cell type-specific reference datasets (Additional file 1: Figure S6 and Table S9) and accounting for the RF+ and CCP+ status (that showed consistent association with response to anti-TNF treatment in both cohorts—Table 1) of the study subjects in the model (Additional file 1: Table S10). The cell type-specific analysis was performed using five comparable publicly available RA datasets containing pre-treatment gene expression data in blood for EULAR-GR and EULAR-NR to anti-TNF therapy [17–19, 32, 33]. Three of the five datasets display qualitatively similar results with, on average, higher expression levels of genes elevated in the innate compartment in EULAR-GR at baseline and higher levels of the genes elevated in the adaptive compartment in EULAR-NR at baseline (Fig. 4b, Additional file 1: Table S12).

An additional finding which may be related to innate immune cells, likely neutrophil activity, is the potential association between the functionally significant NA1 and NA2 FcγRIIIb gene allele-linked glycopeptide variants and RA patient response to anti-TNF therapy. FcγRIIIb genotype counts (excluding one subject per cohort carrying rare SH allele) by EULAR response status for each of the two cohorts (Additional file 1: Table S13) showed statistically significant association between genotype and response in C1 (*p* = 0.01), but not in C2 (*p* = 0.9). The significance estimate for the analysis of the data pooled between two cohorts is *p* = 0.07.

### Influence of baseline innate/adaptive balance on treatment response

**Fig. 5:**
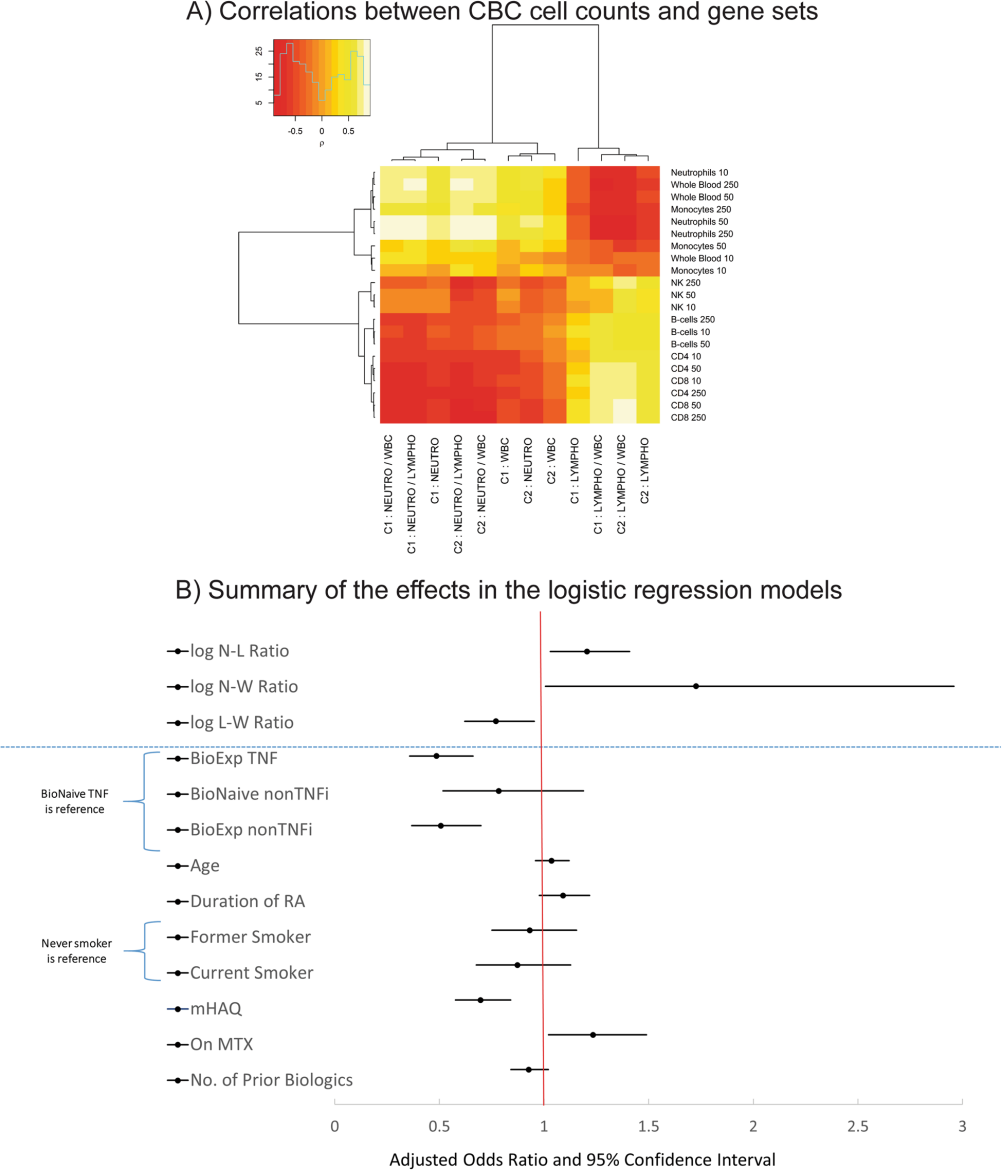
**a** Spearman correlations between CBC metrics (counts of neutrophils, lymphocytes, and their ratios) and average gene expression levels for gene sets predominantly expressed in major immune cell types. **b** Forest plot representation of the effects of baseline patient attributes on the probability of good or moderate response at 3 months follow-up. Odds ratios (ORs) for cell count log-ratios are estimated by three models using each of these log-ratios respectively in addition to the rest of the covariates shown below the blue dashed line. Representative ORs for the rest of the attributes are from the model including neutrophil-to-lymphocyte log-ratio as a covariate. OR for N-L, N-W, and L-W log-ratios are per 1 unit increase in natural logarithm of the corresponding ratio. OR for mHAQ is for 1 unit increase in mHAQ. OR for no. of prior biologics is for each additional prior biologic used. OR for age and duration of RA is for a 10-year increase in age and duration of RA, respectively, from the N-L ratio model. See main text for further details

Genes overexpressed in innate/adaptive immune cell types showed higher correlation with ratios of selected CBC metrics than with their untransformed values (Fig. 5a, Additional file 1: Figure S7). Logistic regression models were developed to evaluate the probability of good or moderate treatment response (based on EULAR criteria) at MO3, as a function of baseline CBC neutrophil-to-lymphocyte (NLR), neutrophil-to-WBC (NWR), or lymphocyte-to-WBC (LWR) log-ratios. Models were established for the remaining ~ 2000 patients of the entire CERTAIN registry (including also biologic-experienced subjects and non-TNFi initiations) that had baseline neutrophil, lymphocyte, and WBC measurements, and known EULAR response at MO3. They were either evaluated without adjustment, or by adjusting for covariates such as the type of biologic (TNFi or non-TNFi), age, disease duration, smoking status, modified HAQ, concomitant MTX treatment, and number of prior biologics. Figure 5b depicts the odds ratios of good or moderate response for CBC log-ratios and other covariates (from the model including NLR). By this model, a one-unit increase in baseline NLR log-ratio resulted in a 1.23 increased probability of good or moderate response (unadjusted OR = 1.23, 95% CI = [1.06, 1.42], *p* = 0.007; adjusted OR = 1.20, 95% CI = [1.03, 1.41], *p* = 0.02). The effect is comparable to that of concomitant MTX treatment (adjusted OR = 1.23, 95% CI = [1.02, 1.49], *p* = 0.03), which is used as a first-line therapy. Similarly, a one-unit increase in baseline NWR log-ratio resulted in a 1.9 increased probability of good or moderate response (unadjusted OR = 1.91, 95% CI = [1.14, 3.18], *p* = 0.01; adjusted OR = 1.73, 95% CI = [1.01, 2.96], *p* = 0.05). Conversely, the association between increased lymphocytes and non-response is emphasized by a 24% decreased probability of good or moderate response, following a one-unit increase in baseline LWR log-ratio (unadjusted OR = 0.76, 95% CI = [0.62, 0.93], *p* = 0.007; adjusted OR = 0.77, 95% CI = [0.62, 0.95], *p* = 0.02). These results suggest that CBC readouts at baseline, either as neutrophil-to-lymphocyte ratio, or as normalized lymphocyte or neutrophil counts (i.e., lymphocyte/WBC and neutrophil/WBC), can be useful to assess the predictability of response to anti-TNF treatment in RA patients. However, despite a statistically significant association between measurements of immune/adaptive cell type composition of blood samples and the probability of those RA patients to respond to treatment by biologic agents, their impact on the predictive performance of the resulting model was negligible (∆AUC < 0.01).

## Discussion

This report describes comprehensive molecular profiling of RA patients undergoing anti-TNF therapy. Transcriptomics analysis confirmed a reduction of inflammatory pathways, with a marked decrease of myeloid-specific functions following 12 weeks of anti-TNF treatment in two independent cohorts. Conversely, markers of adaptive immune functions, including T cell markers and protein synthesis, were increased, which may be related to the overall decrease in myeloid transcripts due to relative nature of transcriptomics measurements. The modulation of migratory and chemotactic processes might explain the decrease in neutrophil markers. Engagement of membrane TNF (mTNF), which is expressed on neutrophils and is associated with induction of apoptosis, might also account for this reduction [34].

Proteomics analysis also showed a reduction in pro-inflammatory markers including complement and acute phase proteins. Changes following anti-TNF treatment were highly correlated between EULAR-GR and EULAR-NR in gene expression and proteomics data. Concordance of molecular changes following anti-TNF treatment for EULAR-GR and EULAR-NR was also reported in an earlier study [35] and further confirmed by the analysis of a publicly available dataset [33]. Collectively, these results suggest that distinct pharmacodynamic effects of anti-TNF treatment are observed in most patients, but are not necessarily associated with clinical response.

At baseline, innate immune cell type-specific genes were on average expressed at a higher level in EULAR-GR from both cohorts, while the adaptive immune cell type-specific genes were on average elevated in EULAR-NR. This observation was confirmed in three publicly available datasets [17, 18, 33], to which we applied the cell type-specific gene expression analysis. The reproducibility of this association, despite the differences among those studies, including patient selection and characterization, indicates that the innate/adaptive balance is an important contributor to clinical response to anti-TNF therapy. The ability of myeloid cells to secrete inflammatory cytokines, including TNF, might contribute to the association between inflammation and response.

Analysis of CBC measurements for the remaining ~ 2000 patients of the CERTAIN study confirmed a higher fraction of neutrophils at baseline in good or moderate responders and, conversely, increased levels of lymphocytes at baseline in non-responders. Anti-TNFs, tocilizumab, and abatacept initiations constitute the majority of this cohort suggesting that innate/adaptive immune cell balance may also influence the response to non-TNF agents that have a potential impact on or are influenced by innate immune functions [36–39]. These results utilizing clinical assessment-based hypothesis testing further support the associations from molecular profiling.

The ability of neutrophils to secrete TNF in response to binding of immune complexes to Fc gamma receptors (FcγRs) in the synovial fluid may contribute to joint damage in RA. FcγRs are considered to play a crucial role in RA pathogenesis [40], and various SNPs in FcγRIIa, FcγRIIIa, and FcγRIIIb have been associated to susceptibility to RA [41–43]. While the high homology between the low affinity FcγR genes has challenged the generation of probes for genotyping, quantitation of FcγRIIIb glycopeptides enabled more specific measurements. Results showed a strong association of the NA1/NA1 genotype with non-response in C1. While this association remains close to statistical significance when patients across both cohorts are combined, the absence of this association in C2 suggests potential value in evaluating it across the broader group of patients in order to further understand its contribution to the risk of non-response to anti-TNF treatment.

Multiple studies using molecular profiling of blood samples from RA patients have been conducted to characterize the molecular response to anti-TNF treatment [12–14, 17–19, 32, 33]. While most of them reported identification of genes predictive of response, replication has been challenging. Oswald and colleagues [12] showed no significant difference in immune populations between responders and non-responders to anti-TNF at baseline in three datasets including the ABCoN cohort. Similarly, our cell type-specific approach did not allow discrimination between responders and non-responders at baseline in the ABCoN data, potentially reflecting heterogeneity of transcriptional profiles of RA patients at baseline across multiple studies.

Variability between studies has been ascribed to multiple factors, including patient selection, tissues analyzed, and sample processing. Our study evaluated two cohorts of RA patients selected from the CERTAIN registry with the focus on increasing the comparability of the two cohorts (e.g., restricting the selection to biologic-naïve patients on concomitant MTX and excluding patients with anti-drug antibodies). However, our study conclusions are based on molecular characterization of a limited number of samples representing complex mixtures of different cell types (such as whole blood) from highly heterogeneous patient population (such as RA) and, as such, are inherently subject to several limitations. They include the use of more extreme EULAR-GR and EULAR-NR for molecular characterization and the separation in time of the selection of subjects for C1 and C2 by about 2 years, so that at each instance patient selection was limited to the subjects enrolled in the CERTAIN registry at that time. Therefore, any potential biases due to systematic differences between early vs. late enrolled patients in their clinical and demographic characteristics as well as the differences in patient presentations at clinical practices joining the registry earlier or later can potentially impact reproducibility of the findings between these two cohorts. These concerns are partially alleviated by the assessment of pertinent publicly available data and relying on CBC metrics for the analysis of the remaining ~ 2000 RA patients in CERTAIN registry, but an independent targeted and higher throughput characterization of the relative balance of innate and adaptive compartments in RA patients would enable further elucidation of this effect in conjunction with other clinical and demographic indicators when evaluated across larger patient cohort. Heterogeneity of findings regarding NLR and probability of clinical response to biologic therapies recently reported for smaller cohorts in RA underscores the importance of evaluating this relationship across wider variety of larger patient samples by more targeted approaches [36, 44–46].

The approach of using multiple analytical techniques to characterize RA patients provided useful biological insights into their response to anti-TNF therapy. While genome-wide analysis demonstrated limited reproducibility of the differences between good and non-responders at baseline across the two cohorts, a cell type-specific transcriptional analysis demonstrated the prominent role of innate/adaptive balance not only in response to anti-TNF treatment, but also for differences between EULAR-GR and EULAR-NR at baseline. Second-line biologics, including abatacept (CTLA-4-Ig), rituximab (anti-CD20), and tocilizumab (anti-IL-6R), which target B and T cell responses, have shown increased efficacy in RA patients failing response to a first anti-TNF therapeutic [47–51], further suggesting the involvement of the adaptive compartment in the lack of response to anti-TNF. The statistically significant effect of CBC measurements on the probability of response in the model analyzing therapeutic initiations for all biologics included in the CERTAIN registry suggests potential relevance of this association across various therapeutics for RA. Even though the CBC measurement alone did not cross the threshold for a statistically robust predictor of response to anti-TNF therapy, it should be considered if this measurement, along with additional clinical and laboratory observations, could be useful in the selection of appropriate biologic therapy for the effective management of rheumatoid arthritis.

Such variability in immune cell type composition of RA patients as reflected by gene expression or CBC measurements could be an important factor in modulating their response to anti-TNF and other biologic treatments. More targeted elucidation of molecular differences in the composition of the innate and adaptive compartments across RA patient population could, ultimately, yield better treatment assignments to patients. This study further emphasizes the importance of combining multiple parameters, including clinical observations, genetic and genomic data, in addition to cellular and biochemical data in order to better understand the mechanisms driving response to biologics in RA.

## Conclusions

Through comprehensive molecular profiling, we identified that RA patients receiving anti-TNF therapy in combination with methotrexate for 3 months exhibit a reproducible profile of changes in whole-blood gene expression and plasma proteomics, regardless of their clinical response to therapy. These changes in expression profile are consistent with a decrease in blood neutrophil counts and associated biology. Decreases in blood neutrophil counts were independently confirmed by CBC laboratory measurements. Separately, a difference in baseline immune cell populations in blood was shown to be associated with the probability of therapeutic response following 3 months of treatment, with patients exhibiting an increased frequency of adaptive immune cell signatures being less likely to respond to therapy. We hypothesize that the inability of anti-TNF therapy to suppress adaptive immune-related pathways may contribute to the likelihood of treatment failure in these patients. Similar association to treatment response could be observed across when analyzing multiple previously published studies, as well as for a broader patient cohort characterized by CBC measurements, further substantiating our findings. Additional evaluation of the clinical utility of baseline immune cell phenotyping in assessing potential patient outcomes is warranted.

## Supplementary information

**Additional file 1:** Supplementary methods; **Tables S1**, **S2**, **S7-S13**; **Figures S1-S7.**

**Additional file 2: Tables S3-S6.**

## Data Availability

The datasets generated and/or analyzed during the current study are available in the NCBI-GEO repository [https://www.ncbi.nlm.nih.gov/geo/query/acc.cgi?acc=GSE129705].

## Abbreviations

AUC: Area under true positive vs. false positive rate curve
BH-FDR: Benjamini-Hochberg false discovery rate
BL: Baseline
BMI: Body mass index
C1: Cohort 1
C2: Cohort 2
CBC: Complete blood count
CCP: Cyclic citrullinated peptide
CDAI: Clinical disease activity index
CERTAIN: Comparative Effectiveness Registry to study Therapies for Arthritis and Inflammatory conditions (ClinicalTrials.gov identifier: NCT01625650)
CI: Confidence interval
CRP: C-reactive protein
DAS28: Disease activity score assessing 28 joints
DAS28-CRP: DAS28 including CRP measurements
DMARDs: Disease-modifying anti-rheumatic drugs
EULAR: European League Against Rheumatism
EULAR-GR: EULAR responder
EULAR-NR: EULAR non-responder
Fc: Fragment crystallizable region of antibody
FcγR: Fc gamma receptor
GO: Gene Ontology
HAQ: Health assessment questionnaire
LWR: Lymphocyte-to-WBC ratio
MO3: Month 3
MTX: Methotrexate
NCBI-GEO: National Center for Biotechnology Information Gene Expression Omnibus
NGS: Next-generation sequencing
NLR: Neutrophil-to-lymphocyte ratio
NWR: Neutrophil-to-WBC ratio
OR: Odds ratio
RA: Rheumatoid arthritis
RF: Rheumatoid factor
SJC28: 28 swollen joint count
SNP: Single nucleotide polymorphism
TJC28: 28 tender joint count
TNF: Tumor necrosis factor
TNFi: TNF inhibitor
UCSC: University of California, Santa Cruz
WBC: White blood cells
