# Supplementary material for: Molecular profiling of rheumatoid arthritis patients reveals an association between innate and adaptive cell populations and response to anti-tumor necrosis factor
Source: Arthritis Res Ther. 2019 Oct 23;21:216. doi: 10.1186/s13075-019-1999-3 (PMC6813112; doi:10.1186/s13075-019-1999-3)
Supplement: Supplementary file 1 — Additional file 1: Supplementary methods; Tables S1, S2, S7-S13; Figures S1-S7. [file 13075_2019_1999_MOESM1_ESM.pdf]

Supplementary material: Molecular profiling of rheumatoid arthritis patients reveals an association between innate and adaptive cell populations and response to anti-tumor necrosis factor

Victor Farutin, Thomas Prod'homme, Kevin McConnell, Nathaniel Washburn, Patrick Halvey, Carol J. Etzel, Jamey Guess, Jay Duffner, Kristen Getchell, Robin Meccariello, Bryan Gutierrez, Christopher Honan, Ganlin Zhao, Nicholas A. Cilfone, Nur Sibel Gunay, Jan L. Hillson, David S. DeLuca, Katherine C. Saunders, Dimitrios A. Pappas, Jeffrey D. Greenberg, Joel M. Kremer, Anthony M. Manning, Leona E. Ling and Ishan Capila

27 August, 2019

Contents

1 Supplementary methods **1**

2 Treatment (MO3-BL) effect analysis details **3**

3 Analysis of the baseline differences between good responders and non-responders **7**

4 Cell type signature of EULAR GR-NR differences at baseline **12**

5 FcyRIIIb genotype and response to anti-TNF therapy **15**

6 Correlation between gene sets and blood cell counts **15**

Session Info **17**

Supplementary References **17**

1 Supplementary methods

1.1 Plasma sample processing

Plasma ID numbers were randomly assigned to all plasma samples. Samples were processed in the order of plasma ID numbers to insure minimal bias due to run order, and run as sets of 20 samples. A normal human plasma control (obtained from Sigma-Aldrich) was included with each set. Plasma samples were first depleted of the top 14 most abundant proteins using Multiple Affinity Removal System 14 (MARS-14), an immunoaffinity, HPLC-based methodology, allowing for the detection of medium to low abundant proteins. Protein concentration was determined using BCA.

1.2 Proteomics analysis by LC/MS-MS

50 µg of total protein was digested using Trypsin/Lys-C. Peptide mixtures were separated using an Ultimate 3000 RSLC nano system. Peptides were loaded onto an Acclaim PepMap RSLC Nano trap column (5 µm particle size, 20 mm x 100 um) at 5 µL/min flow rate and resolved on the basis of hydrophobicity using an EASY-Spray Acclaim PepMap RSLC C18 column. MS analyses were performed on Orbitrap Velos Pro in the positive-ion mode using an EASY-Spray nano-source. RAW files from the mass spectrometer were searched using Sequest HT as part of Proteome Discoverer 1.4 mass informatics software package. Files were searched against the human Uniprot database and then saved as a multiconcensus report (5% peptide-level false discovery rate).

1.3 RNA preparation and NGS sequencing (RNA-seq)

RNA was extracted from human whole blood samples preserved in PAXgene tubes (Qiagen). RNA extraction was performed according to the PAXGene Blood miRNA kit protocol (C1) or PAXGene Blood RNA kit protocol (C2) using the QIAcube instrument (Qiagen). RNA concentration was measured by absorbance at 260nm, and RNA quality was measured by the Agilent TapeStation and Agilent Bioanalyzer. Libraries were prepared for RNA-seq analysis with the Apollo 324 system from WaferGen Biosystems using the WaferGen Prep-X Directional RNA-Seq kit (C1) or Illumina's TruSeq Stranded mRNA Library Preparation Kit (C2) according to manufacturer's protocols. Libraries were sequenced on an Illumina HiSeq 2500 for 40 x 40 bases (C1), and 75 x 75 bases (C2), in paired end, high output mode.

1.4 Determination of plasma levels of adalimumab or infliximab

Plasma levels of adalimumab and infliximab were determined using a sandwich ELISA assay and a standard curve generated by adding exogenous adalimumab or infliximab to human plasma. Adalimumab or infliximab were spiked into human plasma and serially diluted (1:2) for a total of 11 points starting from 200 ng/mL or 400 ng/mL respectively. Pure plasma was used for the zero concentration. Patient plasma samples (100 uL), from the 3-month time point post initiating anti-TNFa therapy, were used in the appropriate ELISA depending on the drug received by the patient. Plates were coated with recombinant human TNFa (R&D System).

For quantification of adalimumab levels, an affinity purified rabbit anti-adalimumab IgG (Maine Biotechnology Services) was used as the detection antibody. A donkey anti-rabbit IgG(H+L)-HRP antibody (Jackson Immuno Research Lab) was used as the secondary antibody.

For quantification of infliximab levels, a human anti-infliximab antibody (HCA213; Bio-Rad) was used as the detection antibody. A donkey anti-human IgG(H+L)-HRP antibody (Jackson Immuno Research Lab) was used as the secondary antibody.

Both assays used TMB as a substrate (Thermo Scientific) for color development, and plates were read at 450 nm / 650 nm after adding the stop solution at a pre-determined time.

1.5 Data preprocessing notes

Only RNA-seq data for genes with median read count of 100 reads or more for each cohort were used for statistical analyses. Differential expression analysis was performed using "voom" modification of "limma" to log-transform and quantile-quantile normalize the data and to account for the dependency between variance and mean of gene expression levels (1,2).

For proteomics data only peptides that were identified by at least 2 PSMs in every sample were used for statistical analyses in each cohort. Additionally, 0 samples from the 1st and 3 samples from the 2nd cohort with fewer than 10^4 total PSMs were excluded from the differential expression analyses (resulting in ranges of total PSM counts of 13909 - 20342 for the 1st and 12586 - 18920 for the 2nd cohort). Shotgun proteomics PSM

Table S1: Demographic and clinical attributes of all subjects in the two cohorts (C1 and C2) that have available RNA-seq data at the baseline. GR and NR indicate EULAR good responders and non-responders respectively.

	C1: GR	C1: NR	p(C1)	C2: GR	C2: NR	p(C2)
N	18	16	N/A	18	11	N/A
Female, N(%)	14(78)	14(88)	0.77	14(78)	8(73)	1
Age, mean(SD)	53(12)	56(13)	0.41	55(12)	48(9.1)	0.14
White, N(%)	16(89)	10(62)	0.16	16(89)	10(91)	1
BMI, mean(SD)	29(7.9)	30(7)	0.51	30(7.3)	33(7.3)	0.2
College educated, N(%)	10(56)	9(56)	1	10(56)	8(73)	0.6
Non-smoker, N(%)	7(39)	11(69)	0.16	11(61)	7(64)	1
Current or previous smoker, N(%)	11(61)	5(31)	0.16	7(39)	4(36)	1
Remicade, N(%)	8(44)	7(44)	1	5(28)	5(45)	0.57
Humira, N(%)	10(56)	9(56)	1	13(72)	6(55)	0.57
SJC28 [BL], mean(SD)	6.7(3.8)	8.7(5.3)	0.25	9.7(5.8)	9.6(5.4)	0.87
TJC28 [BL], mean(SD)	8.7(6.2)	14(8.3)	0.072	12(7.1)	15(5.1)	0.11
ln(CRP) [BL], mean(SD)	1.5(1.6)	1.3(1.9)	0.69	1.7(1.4)	1.5(1.2)	0.81
DAS28CRP [BL], mean(SD)	4.5(0.75)	5.2(0.95)	0.012	4.8(0.87)	5.4(0.58)	0.056
RA duration, mean(SD)	3.8(3.4)	1.8(1.7)	0.04	5.1(6.9)	8.6(9.2)	0.39
RF+, N(%)	15(83)	8(50)	0.088	14(78)	6(55)	0.37
CCP+, N(%)	15(83)	6(38)	0.017	16(89)	5(45)	0.035

data, similarly to gene expression fragment count data, manifests dependency between mean and variance of protein expression levels. In order to account for this dependency limma-voom methodology (1,2) with quantile-quantile normalization was used for the differential expression analyses of shotgun proteomics data as well.

Publicly available gene expression data was downloaded from NCBI-GEO using R/Bioconductor package `GEOquery` (3), quantile-quantile normalized and mapped to Entrez gene symbols using annotation available in Bioconductor (4) or, otherwise, as provided by NCBI-GEO (5).

All correlations reported below are Spearman's rank-based. Their statistical significance was estimated using 3000 permutations. In order to preserve the correlation structure of the data, each permutation involved randomization of the sample labels representing the comparison of interest (i.e. baseline vs. month 3 follow-up or EULAR good responders vs. non-responders, depending on the problem), followed by limma-voom analysis using resulting permuted model matrix.

2 Treatment (MO3-BL) effect analysis details

2.1 Adjusting for variability in RNA-SeQC metrics

Variability related to RNA extraction, library preparation, sequencing batches, etc. was accounted for by adding principal components of QC metrics to the linear model created in limma-voom. QC metrics were produced by RNA-SeQC (6), then centered and scaled before deriving the components. Table S2 shows the associations between gene expression and top PCs of QC metrics by reporting the percentages of genes passing several FDR thresholds. The p-values used to derive the Benjamini-Hochberg adjusted FDR were calculated using a limma-voom model which also accounted for study subject effect and difference between baseline and follow-up samples.

For the analysis of the treatment (MO3-BL) effect gene expression values were adjusted by the inclusion of the corresponding first principal components of scaled RNA-SeQC metrics from each cohort due to their

Table S2: Percentages of genes associated with the top five principal components of centered and scaled RNA-SeQC metrics at several thresholds on BH-FDR in the analysis of the treatment effect

study	QCPC	1%	5%	10%	study	QCPC	1%	5%	10%
C1	QCPC1	32	48	57	C2	QCPC1	3.4	15	27
C1	QCPC2	0	0	0	C2	QCPC2	0	0	0
C1	QCPC3	0	0	0	C2	QCPC3	0	0	0
C1	QCPC4	0	0	0	C2	QCPC4	0	0	0
C1	QCPC5	0	0	0	C2	QCPC5	0	0	0.032

markedly greater association with gene expression data.

2.2 Comparison of MO3 (follow-up) and BL (baseline) gene expression levels

Only data for the subjects that have RNA-seq data both for baseline and follow-up was used. Besides the term representing follow-up (month 3) and baseline samples, linear model used by limma-voom additionally included terms for each study subject (to account for between subject variability) and principal component(s) of RNA-SeQC metrics (to account for technical variability associated with RNA extraction, library construction and NGS sequencing) as defined above. For each gene in the dataset, corresponding p-value for the model term representing MO3-BL difference was used as the measure of the statistical significance of the average difference between follow-up and baseline gene expression levels. Genome-wide statistical significance of MO3-BL differences in gene expression levels was evaluated as the percentage of such p-values passing threshold of 0.05 on corresponding BH-FDR values. Rank-based (Spearman) correlation between corresponding model coefficients representing MO3-BL differences (on log base 2 scale) across all genes included in the analyses both in C1 and C2 was used to assess permutation-based significance of the concordance of such differences between two cohorts. Table S3 in Supplementary Excel workbook `Additional_File_2.xlsx` displays genes with $|\log 2(MO3/BL)| > 0.5$ both in C1 and C2. Table S4 in Supplementary Excel workbook `Additional_File_2.xlsx` displays Gene Ontology categories (7,8) passing BH-FDR cutoff of 0.01 after being scored using `cameraPR` functionality in R/Bioconductor package `limma` with the treatment effect (MO3-BL) in gene expression data averaged between the two cohorts.

2.3 Treatment effect on genes overexpressed in immune cell types

Figure S1 presents comparison of treatment (follow-up vs. baseline) effects in C1 and C2 to the extent of gene expression in various immune cell types (besides neutrophils shown in the main text Figure 1C of the manuscript) and whole blood in NCBI-GEO dataset GSE60424 on immune cell type profiling by RNA-seq (9). In general, in both cohorts the increase in average gene expression levels at the follow-up comparing to the baseline tends to be positively correlated with their overexpression in immune cell types representing adaptive compartment (e.g. CD4 and CD8 T cells, B cells and NK cells) comparing to the rest of sample types in Benaroya dataset even if unremarkable by permutation controls. Table S5 in Supplementary Excel workbook `Additional_File_2.xlsx` displays the top 100 of the genes that are overexpressed in the neutrophils that make the largest contributions to the significance of the negative correlation between their expression levels in the neutrophils and average treatment effect (MO3-BL) in C1 and C2. The contribution of every gene to this correlation was calculated as the negative difference between the original correlation (for the entire set of 8786 genes present in C1, C2 and GSE60424 datasets) and its value recalculated upon exclusion of each gene one at a time. The values of “log2FCneu”, “MO3BLlog2C1” and “MO3BLlog2C2” shown in Table S5 fall outside of the ranges of corresponding axes in the main text Figure 1C as these points are outside of the corresponding outermost density contours.

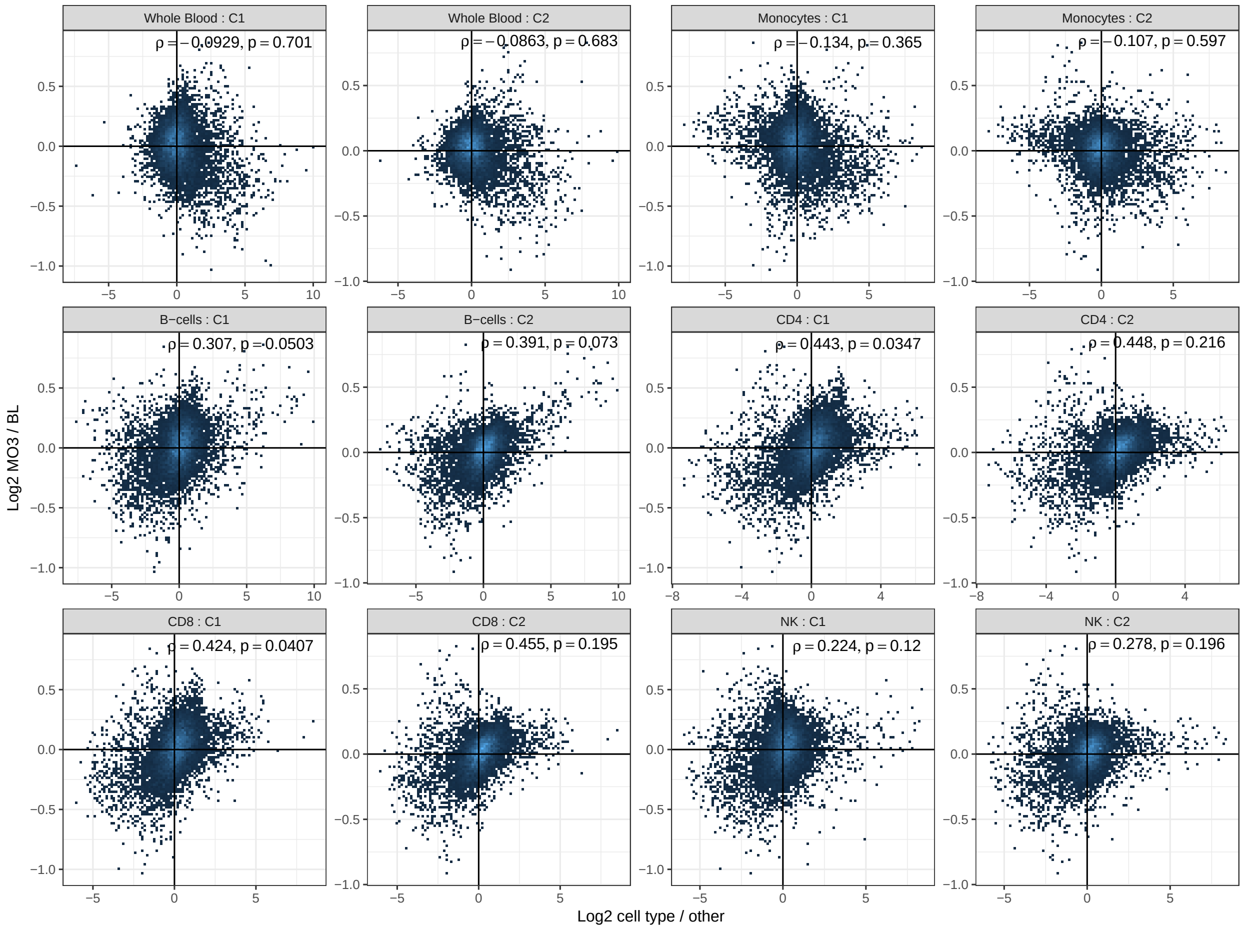

Figure S1: Correlations between log2(MO3/BL) treatment effect in two cohorts (C1 and C2) and gene expression levels in each immune cell type in NCBI-GEO dataset GSE60424.

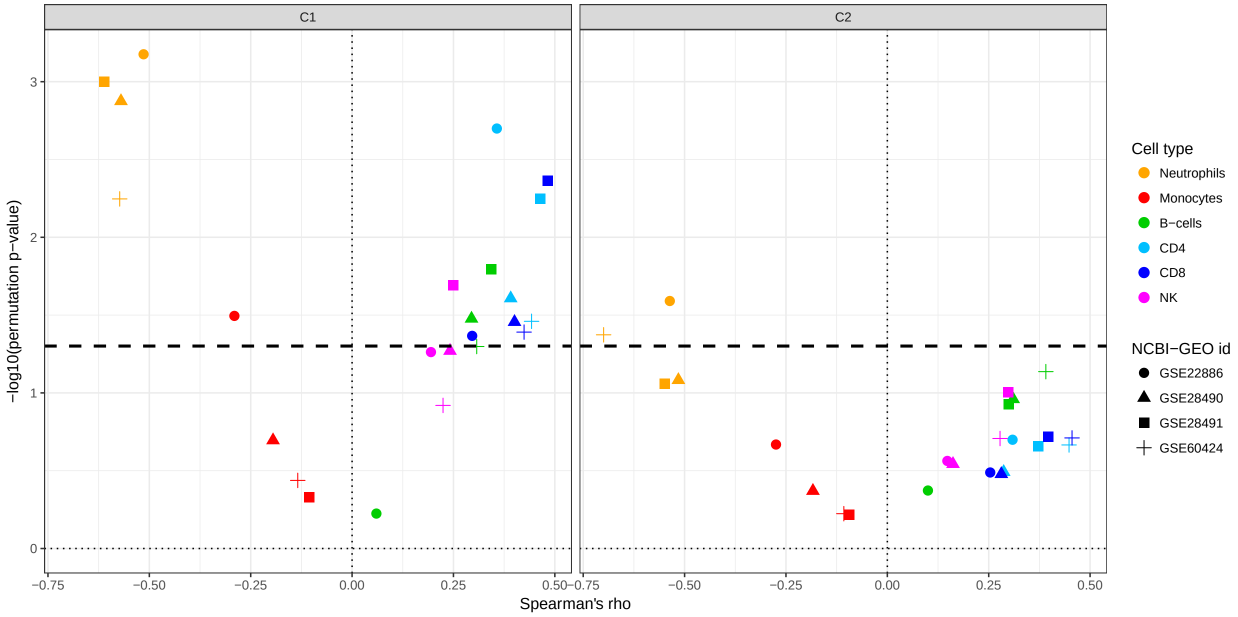

Figure S2: Correlations between anti-TNF treatment effect in C1 and C2 and genes overexpressed in each immune cell type in RNA-seq (GSE60424) and microarrays based (GSE22886, GSE28490 and GSE28491) public datasets profiling major immune cell types. Horizontal dashes indicate p=0.05.

2.3.1 Comparison of treatment effect to cell type specific genes derived from previous RNA-seq and microarray studies

Figure S2 summarizes in the form of volcano plot the results of comparing the gene expression differences following anti-TNF therapy in the two cohorts studied here to the extent of upregulation of these genes in corresponding immune cell types as characterized by some of the microarray-based datasets available in NCBI-GEO (GSE22886 (10), GSE28490 (11), GSE28491 (11)). Previously used NCBI-GEO dataset GSE60424 characterizing major immune cell types by RNA-seq is also included for reference. Regardless of technology used to characterize gene expression in major immune cell types, the conclusions are highly similar: genes most overexpressed in neutrophils and monocytes tend to be downregulated following treatment with anti-TNF and genes overexpressed in adaptive compartment of immune system (B, T and NK cells) are on average upregulated after 3 months on anti-TNF therapy. Permutation based controls reveal varying degree of statistical significance of such associaton. The data from the first cohort, in particular for neutrophils, shows higher levels of significance.

2.4 Treatment effect in plasma shotgun proteomics

Keeping only subjects with both baseline and follow-up samples available after removal of low coverage (fewer than 10^4 total PSMs) samples resulted in 40 and 34 subjects included in the analysis for C1 and C2 respectively. Using more reliably quantified proteins with at least 2 PSMs detected in 100% of samples in each cohort resulted in 159 and 181 proteins analyzed for C1 and C2 respectively. Adjustment for the dependency of variability in PSM counts on their average abundance was performed by adapting 'voom' approach as implemented in 'limma' Bioconductor package. Additionally, the effect of the order in which randomized samples have been quantified by LC-MS measurements has been accounted for in the model by adding the term representing their run order. Between-subject variability has been accounted for by adding to the model categorical variable representing study subjects, so that the effect of anti-TNF treatment represents average within subject follow-up minus baseline difference after adjusting for run order effect. Significance

Table S7: Correlations between treatment effects in good responders and non-responders in Mesko et al. (NCBI-GEO GSE42296) dataset and the two cohorts characterized in this study

GroupA	GroupB	Ngenes	rho	p
MO3-BL C1 Good responder	MO3-BL GSE42296 R - responder	8773	0.367	0.009
MO3-BL C1 Non-responder	MO3-BL GSE42296 R - responder	8773	0.240	0.125
MO3-BL C2 Good responder	MO3-BL GSE42296 R - responder	8300	0.437	0.025
MO3-BL C2 Non-responder	MO3-BL GSE42296 R - responder	8300	-0.001	0.994
MO3-BL C1 Good responder	MO3-BL GSE42296 NR - non-responder	8773	0.366	0.003
MO3-BL C1 Non-responder	MO3-BL GSE42296 NR - non-responder	8773	0.227	0.115
MO3-BL C2 Good responder	MO3-BL GSE42296 NR - non-responder	8300	0.330	0.035
MO3-BL C2 Non-responder	MO3-BL GSE42296 NR - non-responder	8300	-0.021	0.886

estimates for the observed correlation of the effect of anti-TNF therapy between two cohorts and for the counts of proteins with low BH-FDR values in each cohort have been obtained using 3000 permutations randomizing baseline / follow-up status within each subject. Table S6 in Supplementary Excel workbook `Additional_File_2.xlsx` shows Gene Ontology categories passing BH-FDR cutoff of 0.2 after being scored using `cameraPR` functionality from R/Bioconductor package `limma` with the treatment (MO3-BL) effect in proteomics data averaged between the two cohorts.

Relaxing the filter on protein abundance to require at least 1 PSM detected in at least 50% of the samples in each cohort increases the number of proteins passing these cutoffs in both studies to 337. Possibly as a result of including less reliably quantified proteins Spearman correlation between treatment effects in C1 and C2 decreases to 0.12. This larger set of proteins now includes well recognized "positive" acute phase proteins such as C-reactive protein (CRP) and haptoglobin (HP) both down-regulated at follow-up by at least 30% and achieving low p-values in both cohorts.

2.5 Treatment effect in good responders and non-responders in public datasets

NCBI-GEO dataset GSE42296 (12) appears to be the one with the largest number of publicly available gene expression samples for rheumatoid arthritis subjects *both* in responder and non-responder categories including *both* samples obtained before and after anti-TNF therapy. Treatment effect in this dataset is positively correlated between responders and non-responders (Figure S4) and each of them is positively correlated with treatment effect in good responders in both C1 and C2 ($p < 0.05$, Table S7). The effect of anti-TNF therapy in non-responders from C1 is also positively correlated with the effect of anti-TNF treatment in both responders and non-responders in GSE42296 dataset, although this magnitude of correlation coefficient is observed in >10% of random permutation controls. Non-responders from C2 do not show any noteworthy correlation of the effect of anti-TNF therapy with those observed in GSE42296 data. The *differences* between the effects of treatment for responders and non-responders in NCBI-GEO dataset GSE42296 did not attain statistical significance under permutation controls neither at the level of individual genes, nor Gene Ontology categories (BH-FDR ≥ 0.82).

3 Analysis of the baseline differences between good responders and non-responders

3.1 Adjusting for variability in RNA-SeQC metrics

Similarly to the choice of principal components for adjusting to the effects of RNA-seq data preparation and processing for the analysis of effect of anti-TNF therapy treatment (follow-up vs. baseline) described above (Section 2.1), association of baseline gene expression data with corresponding principal components

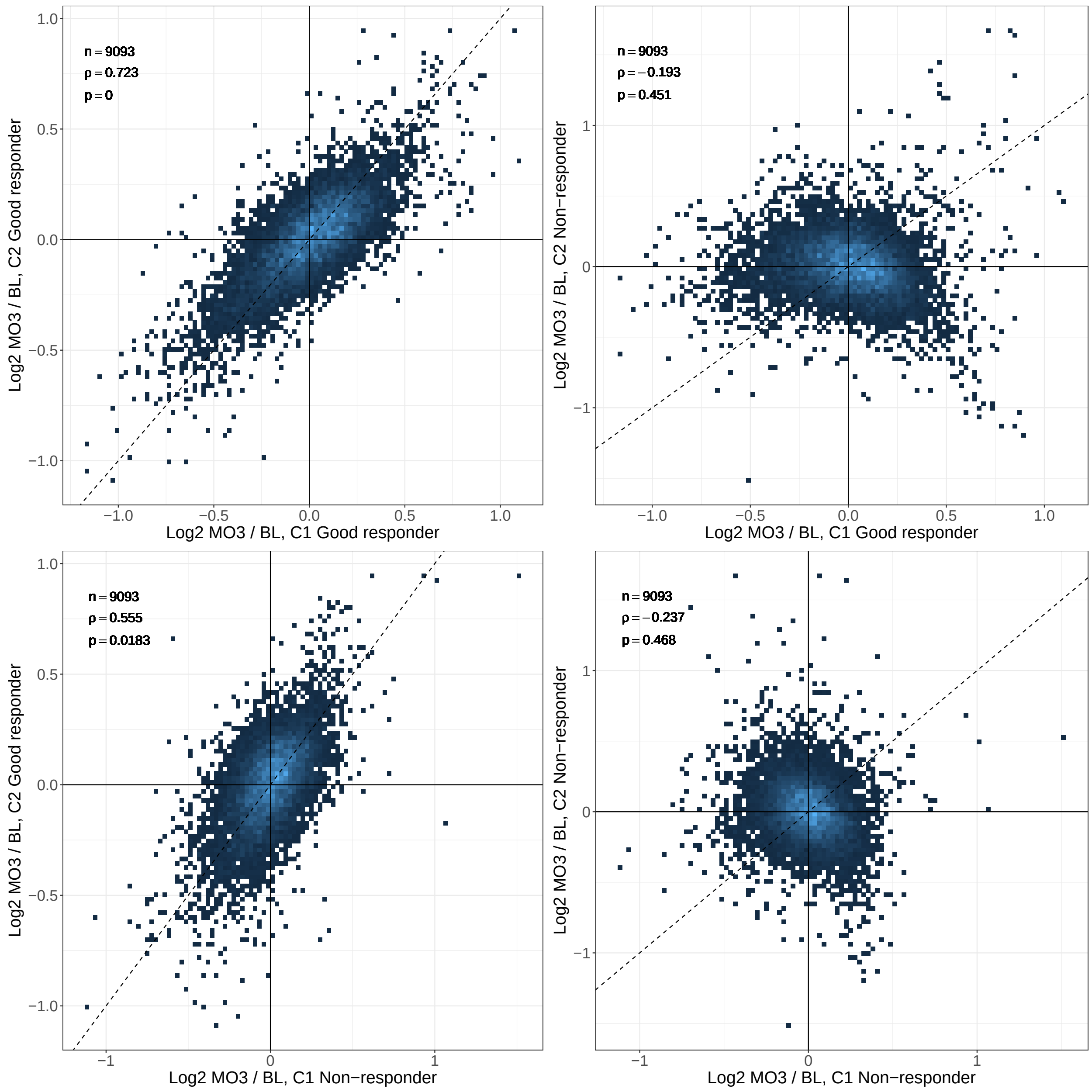

Figure S3: Correlations of treatment effects (log2(MO3/BL) differences) in good responders and non-responders between the two cohorts (supplementing Figure 2A in the main text).

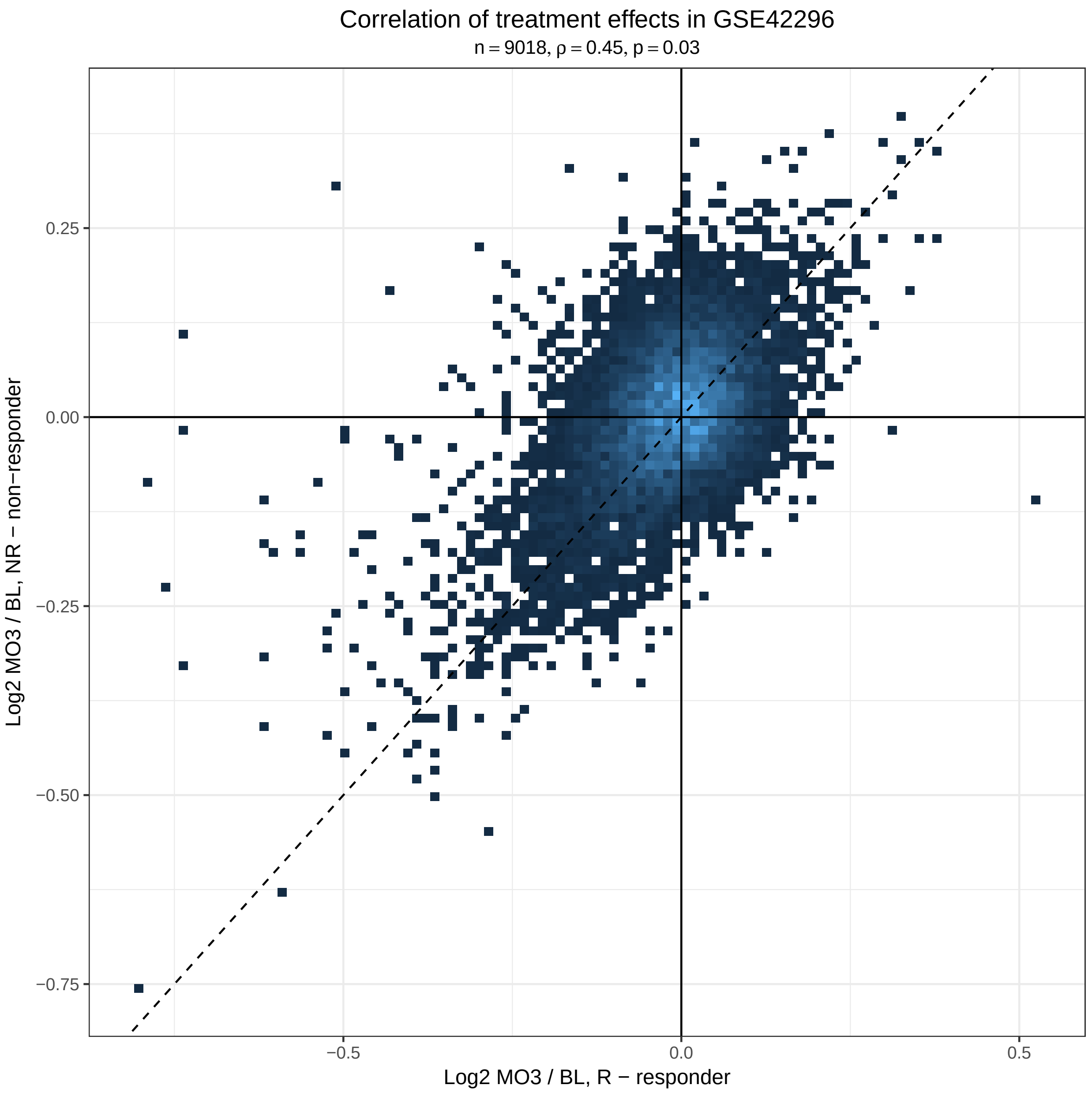

Figure S4: Positive correlation between log2(MO3/BL) differences in responders and non-responders in NCBI-GEO GSE42296 dataset (calculated for genes detected in C1 and/or C2 RNA-seq data as a proxy for being more reliably measurable in whole blood samples).

Table S8: Percentages of genes associated with the top five principal components of centered and scaled RNA-SeQC metrics at several thresholds on BH-FDR in the analysis of the EULAR GR-NR differences at the baseline

study	QCPC	1%	5%	10%	study	QCPC	1%	5%	10%
C1	QCPC1	19	38	47	C2	QCPC1	30	47	56
C1	QCPC2	0	0	0	C2	QCPC2	14	28	37
C1	QCPC3	0.01	0.01	3.8	C2	QCPC3	0	0.011	0.011
C1	QCPC4	0.2	1.6	4.6	C2	QCPC4	0	0.13	0.15
C1	QCPC5	0	0	0	C2	QCPC5	0.011	0.011	0.011

in RNA-SeQC metrics was used to determine the number of such principal components to be included in the model fit to the baseline samples only for the analysis of differential gene expression between good responders and non-responders at baseline.

For differential expression analysis of the baseline EULAR GR-NR differences RNA-seq data for C1 will be adjusted for the first principal component of scaled RNA-SeQC metrics and RNA-seq data for C2 will be adjusted by the 1st and 2nd principal components of scaled RNA-SeQC metrics. Table S8 shows that all of them have large impact on gene expression levels.

3.2 Choice of genes with higher variation at baseline

Similarly to filtering genes on abundance (e.g. median read count), genes can be also filtered by their variance after accounting for the dependency between mean and variance similarly to the approach employed in limma-voom analysis pipeline. Here genes are ranked by the residuals of the dependency between square root of standard deviation and mean of log-transformed count per million (CPM) values as modeled by `lowess` function in R. Genes with larger residuals are those that are more variable after accounting for the mean-variance dependency and probably reflecting greater degree of biological variability of those genes (e.g. as a result of differences in the cell type composition of whole blood samples). Results presented below demonstrate that such more variable genes also tend to show greater degree of the concordance of the EULAR GR-NR differences between the two cohorts.

3.3 Differential expression analysis of EULAR GR-NR effect at baseline

All subjects with baseline RNA-seq data available were included in the analysis (regardless of the availability of matching RNA-seq data at follow-up). In addition to the indicator of good vs. non-responders linear model used in limma-voom also included principal components of RNA-SeQC metrics determined as described above. The model was fit to RNA-seq data using limma-voom procedure. Statistical significance of the EULAR GR-NR differences at the baseline was represented by the p-value corresponding to the model term indicating good response or non-response. Genome-wide significance of these differences was assessed by the number of the genes passing several cutoffs on BH-FDR estimates for corresponding p-values. The rank-based (Spearman) correlation between corresponding model coefficients between the two cohorts was moderately positive, but unremarkable with respect to the permutation controls.

3.4 Correlation and significance for more variable genes

Figure S5 illustrates the impact of the variability of genes at the baseline on the correlation (and associated significance with respect to permutation controls) between model coefficients in the two cohorts representing difference in gene expression data at baseline between good responders and non-responders. More variable genes show greater degree of correlation of this effect between the two cohorts.

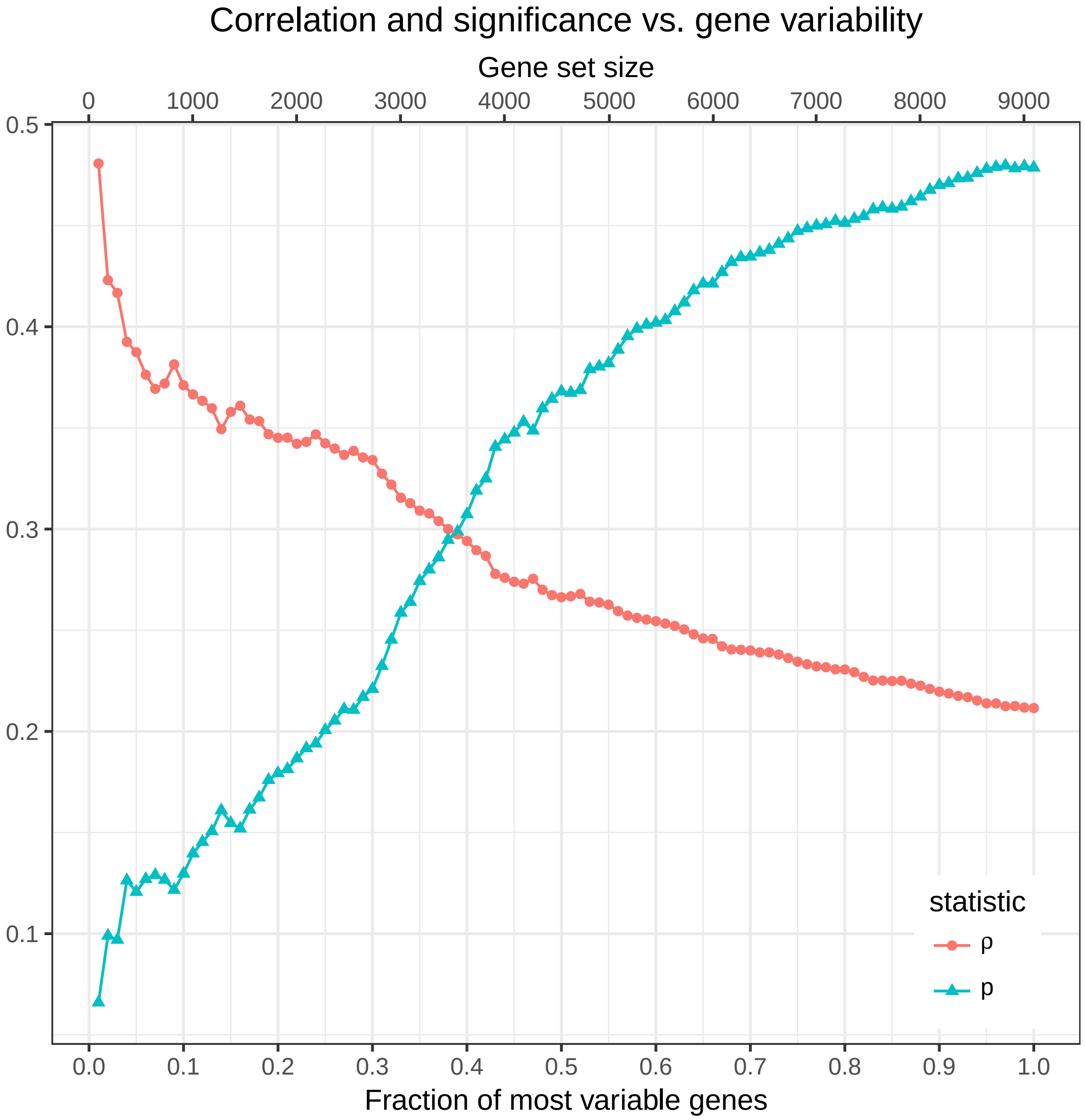

Figure S5: Correlation and significance of the correlation between EULAR GR-NR differences in baseline gene expression data in two cohorts as a function of variability of the genes at the baseline.

Table S9: Permutation based estimates of the two-sided (by absolute value) significance (column "Pvalue") of the average difference (column "aveTmod") between mean moderated t-statistic for EULAR GR-NR effect at baseline for innate and adaptive gene sets (with 10, 50 and 250 genes most overexpressed in each cell type) pooled over two cohorts studied herein

CellTypeRef	Ngenes	aveTmod	Pvalue	CellTypeRef	Ngenes	aveTmod	Pvalue
GSE60424	10	1.67	0.042	GSE28490	10	1.80	0.017
GSE60424	50	1.50	0.045	GSE28490	50	1.40	0.054
GSE60424	250	1.32	0.063	GSE28490	250	1.19	0.075
GSE22886	10	1.85	0.015	GSE28491	10	1.77	0.023
GSE22886	50	1.52	0.035	GSE28491	50	1.41	0.061
GSE22886	250	1.20	0.053	GSE28491	250	1.16	0.080

4 Cell type signature of EULAR GR-NR differences at baseline

4.1 Cell type signature of baseline EULAR GR-NR differences in RNA-seq data from two cohorts

In the absence of significant genome-wide concordance of the differences between good and non-responders at the baseline for the two cohorts, the averages of these differences (in the form of moderated t-statistics in the limma-voom output) have been evaluated for the top 10, 50 and 250 genes most overexpressed in each major immune cell type in NCBI-GEO dataset GSE60424. Resulting averages and associated significance estimates with respect to permutation controls are shown in the main text (Figure 4A) in the form of volcano plots. The two cohorts show similar pattern of association between these averages of moderated t-statistic values and gene set representation of innate (neutrophils, monocytes) or adaptive (CD4/CD8 T-cells, B-cells, NK cells) compartments. Statistical significances of each of these associations individually (for a given combination of cohort, gene set size and cell type) are unremarkable. Average difference between average moderated t-statistic of EULAR GR-NR effect for innate vs. adaptive cell types across the two cohorts is relatively infrequently observed for permutation controls regardless of the size of gene sets that were evaluated here (CellTypeRef=GSE60424 in Table S9).

4.2 Cell type signature results using microarray based cell type datasets

Figure S6 represents the same relationship between baseline gene expression levels in good and non-responders for gene sets overexpressed in major immune cell types using datasets characterizing major immune cell types by microarray as opposed to RNA-seq data (NCBI-GEO GSE22886, GSE28490 and GSE28491). Qualitatively, the conclusion remains unchanged: genes representing the innate compartment of the immune system are on average higher in good responders and, inversely, genes representing the adaptive compartment are higher in non-responders. The significance of average differences in moderated t-statistic for EULAR GR-NR effect between genes overexpressed in immune cell types were derived by permutation and reported in Table S9 (CellTypeRef other than GSE60424).

4.3 Cell type signature results accounting for CCP+ and RF+ status of study subjects

Table S10 represents average differences of moderated t-statistic values between genes overexpressed in innate vs. adaptive immune cell types (from NCBI-GEO dataset GSE60424) for the effects of CCP+ status, RF+ status and EULAR GR-NR estimated by the linear model including as covariates all three of these terms (in addition to the principal components of RNA-SeQC metrics) ("t*" columns) and corresponding estimates of their statistical significance ("p*" columns) obtained by permutation preserving correlation between these

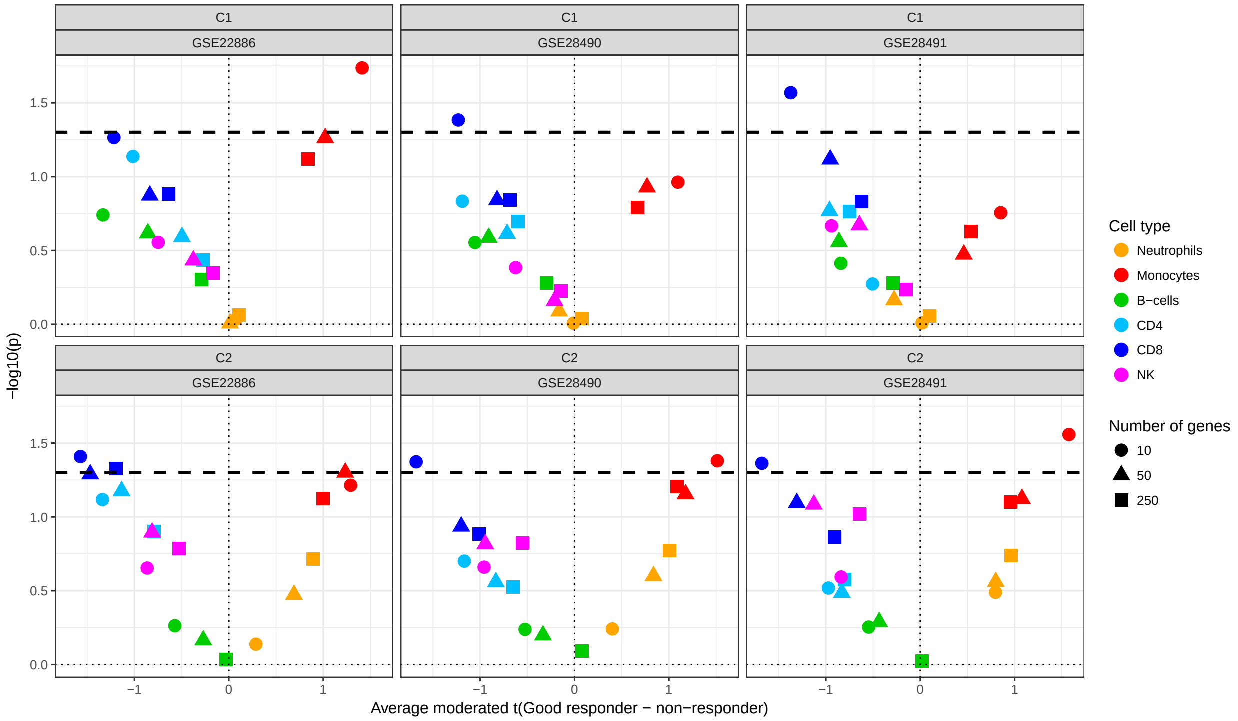

Figure S6: Averages of the EULAR GR-NR differences at the baseline in each cohort for the genes over-expressed in each immune cell type in microarrays-based public datasets profiling major immune cell types (NCBI-GEO ids GSE22886, GSE28490 and GSE28491).

Table S10: Average difference and permutation based estimates of statistical significance for the effects of CCP status, RF status and EULAR response on the innate and adaptive gene sets (as derived from NCBI-GEO dataset GSE60424) in the linear models including all three (CCP, RF and EULAR) covariates. Columns "tCPP", "tRF" and "tEULAR" represent difference between average values of moderated t-statistic values for innate and adaptive gene sets for each of the three effects (CCP, RF and EULAR) in the model. Corresponding columns "pCPP", "pRF" and "pEULAR" represent two-sided (by absolute value) permutation based estimates of their significance.

Ngenes	tCCP	pCCP	tRF	pRF	tEULAR	pEULAR
10	0.66	0.42	-1.30	0.12	1.60	0.05
50	0.65	0.38	-0.90	0.23	1.32	0.07
250	0.75	0.29	-0.83	0.24	1.08	0.12

three attributes in two cohorts in this study. The goal of this evaluation is to assess whether the observed difference between average EULAR GR-NR effects for innate and adaptive immune cell type gene sets at baseline can be readily accounted for by the study subjects' RF+ or CCP+ status which manifested consistent association with clinical response to the anti-TNF treatment in both cohorts (see Table 1 in the main text).

Results presented in Table S10 indicate that the addition of CCP+ and RF+ indicators to the model slightly attenuates the magnitude (column "tEULAR") and significance (column "pEULAR") of the difference in EULAR GR-NR effect between innate and adaptive gene sets (as compared to corresponding values of "aveTmod" and "Pvalue" in Table S9 for CellTypeRef=GSE60424). Nevertheless, the average effect of EULAR GR-NR differences on the expression levels of innate and adaptive gene sets remains the largest by absolute value and the least frequently observed in permutation as compared to those of the attributes indicating CCP+ and RF+ status of study subjects in both cohorts as estimated by the model simultaneously accounting for the effects of all three of these factors.

Lastly, global concordance of the effects of CCP+ and RF+ status on gene expression levels was found to be unremarkable between the two cohorts in this study. The genome-wide correlation of the gene expression differences associated with CCP+ status (after accounting for RNA-SeQC metrics, EULAR GR-NR differences and RF+ status) between C1 and C2 had Spearman coefficient of $\rho = -0.0018$ with corresponding statistical significance of $p = 0.99$ as estimated by permutation. The effects of RF+ status on gene expression levels between C1 and C2 had correlation and significance of $\rho = 0.18$ and $p = 0.57$ respectively.

Summarily, these results suggest that although RF+ and CCP+ status of the study subjects showed consistent association with the clinical response to anti-TNF therapy in the two cohorts evaluated here, their association with gene expression levels is unremarkable and it does not qualitatively impact the observed difference between innate and adaptive cell type gene sets for their average association with the clinical response to treatment.

4.4 Public datasets characterizing response to anti-TNF therapy in rheumatoid arthritis

Table S11 provides high level summary of the five publicly available (NCBI-GEO) datasets characterizing gene expression in blood samples of good responders and non-responders to anti-TNF treatment in rheumatoid arthritis that were analyzed using the aforementioned approach. These datasets were selected as those that had clinical response to anti-TNF treatment in rheumatoid arthritis at about 3 months (e.g. at week 14 for all five datasets analyzed here) determined according to standard clinical criteria (EULAR or ACR) and that had gene expression from a sufficient number of peripheral blood samples measured by commercially established microarray platform (12–16). They were obtained by reviewing the results returned by NCBI-GEO query for GEO datasets: `"rheumatoid arthritis"[All Fields] AND blood[All Fields] AND (anti-TNF[All Fields] OR infliximab[All Fields] OR adalimumab[All Fields] OR etanercept[All Fields] OR golimumab[All Fields]) AND "gse"[Filter] AND "Homo sapiens"[Organism] AND ("Expression profiling by array"[Filter] OR "Expression profiling by high throughput sequencing"[Filter])` to retain those datasets that meet the criteria specified above. The differences between the total count of samples in some of the datasets and those used for the analyses here (fields Total and Used in Table S11) are due to omission of non relevant samples – i.e. moderate EULAR responders in GSE15258, Crohn's disease and follow-up samples in GSE42296 and placebo treated arm and moderate responders in GSE58795.

Application of the same approach for summarizing differences in gene expression data at baseline between good responders and non-responders to anti-TNF therapy for gene sets highly expressed in major immune cell types across these five publicly available gene expression datasets shows pattern similar to the one reported for the two cohorts analyzed in this study for three out of these five datasets. When pooled over all these five datasets (regardless of whether they manifest the pattern of association between innate/adaptive compartment markers at baseline and response to anti-TNF at follow-up or not) such association is infrequently observed in permutation controls regardless of gene set sizes evaluated here or reference immune cell type datasets used to derive gene sets overexpressed in adaptive/innate compartments (Table S12).

Table S11: Gene/sample counts for public datasets in NCBI-GEO characterizing baseline gene expression in whole blood/PBMC/WBC samples from good responders (GR) and non-responders (NR) to anti-TNF treatment for rheumatoid arthritis

GEOID	Probes	Total	Used	Response	Description
GSE12051	17454	44	44	GR=37, NR=7	Julia et al., 2009
GSE15258	54675	86	46	GR=24, NR=22	Bienkowska et al., 2009; GR and NR
GSE33377	21980	42	42	GR=18, NR=24	Toonen et al., 2012
GSE42296	33297	78	19	GR=6, NR=13	Mesko et al., 2013; baseline RA samples
GSE58795	52378	59	12	GR=5, NR=7	MacIsaac et al., 2014; infliximab treated GR and NR

Table S12: Permutation based estimates of the two-sided (by absolute value) significance (column "Pvalue") of the average difference (column "aveTmod") between mean moderated t-statistic for GR-NR effect at baseline for innate and adaptive gene sets (with 10, 50 and 250 genes most overexpressed in each cell type) pooled over five public datasets from NCBI-GEO

CellTypeRef	Ngenes	aveTmod	Pvalue	CellTypeRef	Ngenes	aveTmod	Pvalue
GSE22886	10	0.67	0.108	GSE28490	10	0.92	0.037
GSE22886	50	0.72	0.048	GSE28490	50	0.77	0.053
GSE22886	250	0.56	0.068	GSE28490	250	0.65	0.066
GSE60424	10	0.96	0.033	GSE28491	10	0.94	0.036
GSE60424	50	0.86	0.035	GSE28491	50	0.84	0.038
GSE60424	250	0.66	0.060	GSE28491	250	0.65	0.066

5 FcyRIIIb genotype and response to anti-TNF therapy

Table S13: Counts of EULAR good responders (GR) and non-responders (NR) by the FcyRIIIb genotype in the first (C1) and second (C2) cohort. Significances from Fisher exact test for the association between FcyRIIIb genotype and response to the anti-TNF therapy in the two cohorts and their combination are p(C1) = 0.013, p(C2) = 0.86 and p(C1+C2) = 0.072 respectively

	C1_GR	C1_NR	C2_GR	C2_NR
NA1/NA1	0	5	1	1
NA1/NA2	8	12	14	8
NA2/NA2	10	4	6	5

6 Correlation between gene sets and blood cell counts

Blood cell counts (WBC, lymphocytes, neutrophiles and their ratios) were correlated (Spearman) with the expression of genes which were overexpressed in in neutrophils and CD4/CD8 T-cells. Gene expression was log-transformed, quantile normalized and center-scaled (per gene). The highest correlations were observed with the cell count ratios in comparison to absolute cell counts. Raw (not normalized to account for between subjects variability of CBC metrics) counts of neutrophils, lymphocytes and WBC show markedly lower degree of concordance with gene set averages in RNA-seq data. Figure S7 renders results of clustering of correlation coefficients by their absolute values in the form of a heatmap and indicates that it is normalized blood cell counts that are more correlated with gene set averages.

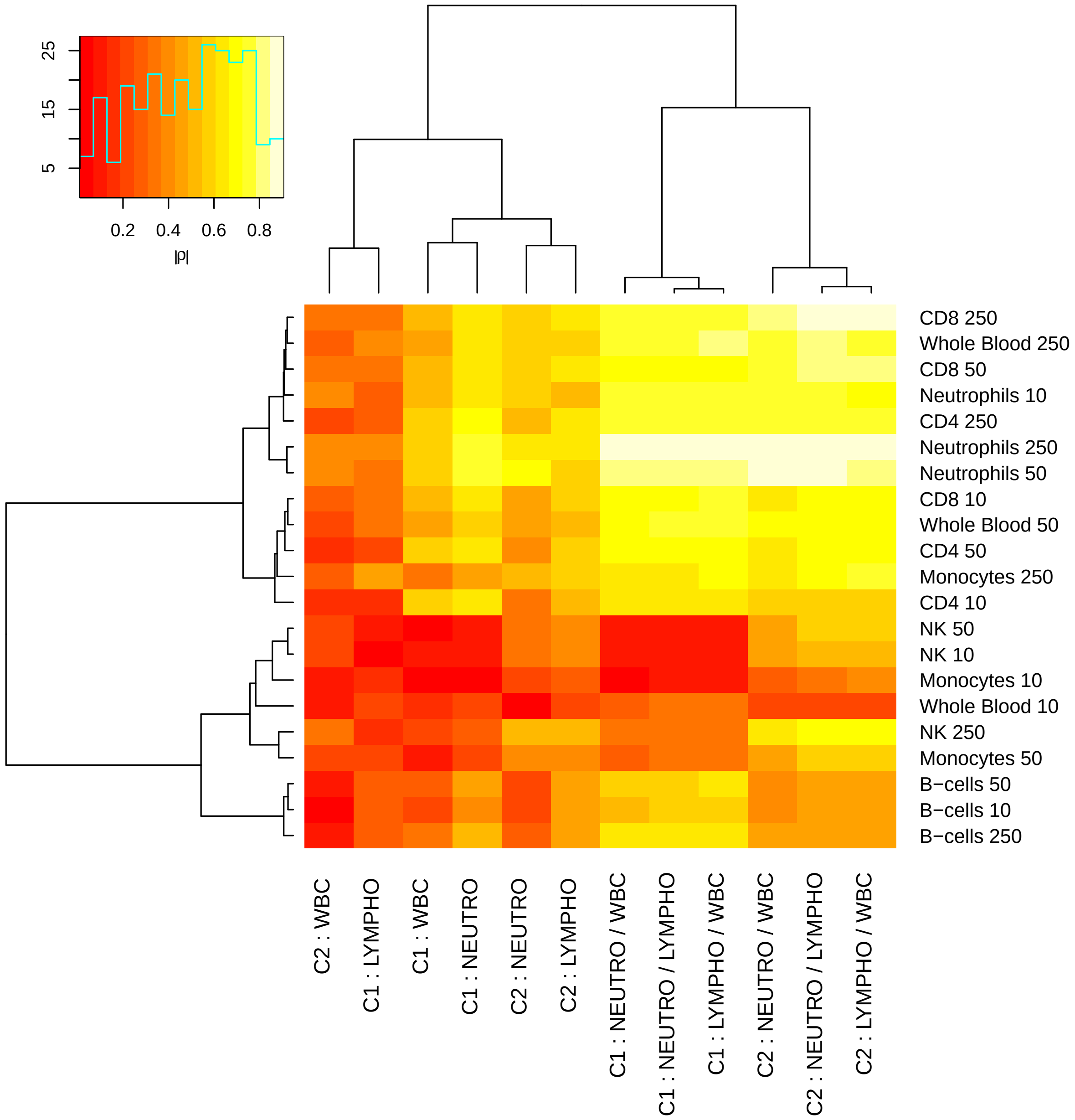

Figure S7: Ward clustering of the absolute values of Spearman correlation coefficients between averages of centered and scaled gene expression levels for immune cell type gene sets and neutrophils (NEUTRO), lymphocytes (LYMPHO) and WBC counts and their ratios from CBC analyses on rheumatoid arthritis patients in C1 and C2.

Session Info

The information below represents versions of R / Bioconductor software used to generate computational results presented above as well as those included in the main text relying on R Markdown/knitr/pandoc capabilities available in R / RStudio.

- R version 3.4.3 (2017-11-30), `x86_64-w64-mingw32`
- Locale: `LC_COLLATE=English_United States.1252`, `LC_CTYPE=English_United States.1252`, `LC_MONETARY=English_United States.1252`, `LC_NUMERIC=C`, `LC_TIME=English_United States.1252`
- Running under: `Windows Server 2008 R2 x64 (build 7601) Service Pack 1`
- Matrix products: default
- Base packages: base, datasets, graphics, grDevices, grid, methods, parallel, stats, stats4, utils
- Other packages: AnnotationDbi 1.40.0, bindrcpp 0.2.2, Biobase 2.38.0, BiocGenerics 0.24.0, doParallel 1.0.11, doRNG 1.7.1, foreach 1.4.4, GEOquery 2.46.15, ggplot2 2.2.1, ggrepel 0.8.0, GO.db 3.5.0, gplots 3.0.1, gridExtra 2.3, hgu133a.db 3.2.3, hgu133b.db 3.2.3, hgu133plus2.db 3.2.3, huex10sttranscriptcluster.db 8.7.0, hugene10sttranscriptcluster.db 8.7.0, illuminaHumanv1.db 1.26.0, illuminaHumanv4.db 1.26.0, IRanges 2.12.0, iterators 1.0.9, kableExtra 0.9.0, limma 3.34.9, lme4 1.1-17, lmerTest 3.0-1, Matrix 1.2-12, org.Hs.eg.db 3.5.0, pkgmaker 0.27, plyr 1.8.4, RColorBrewer 1.1-2, registry 0.5, reshape2 1.4.3, rngtools 1.3.1, S4Vectors 0.16.0, xlsx 0.6.1
- Loaded via a namespace (and not attached): assertthat 0.2.0, backports 1.1.2, bibtex 0.4.2, bindr 0.1.1, bit 1.1-13, bit64 0.9-7, bitops 1.0-6, blob 1.1.1, bookdown 0.7, caTools 1.17.1, codetools 0.2-15, colorspace 1.3-2, compiler 3.4.3, DBI 1.0.0, digest 0.6.14, dplyr 0.7.5, evaluate 0.10.1, gdata 2.18.0, glue 1.2.0, gtable 0.2.0, gtools 3.5.0, highr 0.6, hms 0.4.2, htmltools 0.3.6, httr 1.3.1, KernSmooth 2.23-15, knitr 1.20, labeling 0.3, lattice 0.20-35, lazyeval 0.2.1, magrittr 1.5, MASS 7.3-47, memoise 1.1.0, minqa 1.2.4, munsell 0.4.3, nlme 3.1-131, nloptr 1.0.4, numDeriv 2016.8-1, pillar 1.1.0, pkgconfig 2.0.1, purrr 0.2.5, R6 2.2.2, Rcpp 0.12.17, readr 1.1.1, rJava 0.9-10, rlang 0.2.1, rmarkdown 1.9, rprojroot 1.3-2, RSQLite 2.1.1, rstudioapi 0.7, rvest 0.3.2, scales 0.5.0, splines 3.4.3, stringi 1.1.6, stringr 1.2.0, tibble 1.4.2, tidyr 0.8.1, tidyselect 0.2.4, tools 3.4.3, viridisLite 0.3.0, withr 2.1.2, xfun 0.3, xlsxjars 0.6.1, xml2 1.2.0, xtable 1.8-2, yaml 2.1.19

Supplementary References

1. Law CW, Chen Y, Shi W, Smyth GK. Voom: Precision weights unlock linear model analysis tools for rna-seq read counts. Genome Biology [Internet]. 2014 Feb 03;15(2):R29. Available from: https://doi.org/10.1186/gb-2014-15-2-r29

2. Ritchie ME, Phipson B, Wu D, Hu Y, Law CW, Shi W, et al. Limma powers differential expression analyses for rna-sequencing and microarray studies. Nucleic Acids Research [Internet]. 2015;43(7):e47. Available from: http://dx.doi.org/10.1093/nar/gkv007

3. Davis S, Meltzer PS. GEOquery: A bridge between the gene expression omnibus (geo) and bioconductor. Bioinformatics. 2007;23(14):1846–7.

4. Huber, W., Carey, J. V, Gentleman, R., et al. Orchestrating high-throughput genomic analysis with Bioconductor. Nature Methods [Internet]. 2015;12(2):115–21. Available from: http://www.nature.com/

nmeth/journal/v12/n2/full/nmeth.3252.html

5. Edgar R, Domrachev M, Lash AE. Gene expression omnibus: NCBI gene expression and hybridization array data repository. Nucleic acids research. 2002;30(1):207–10.

6. DeLuca DS, Levin JZ, Sivachenko A, Fennell T, Nazaire M-D, Williams C, et al. RNA-seqc: RNA-seq metrics for quality control and process optimization. Bioinformatics. 2012;28(11):1530–2.

7. Ashburner M, Ball CA, Blake JA, Botstein D, Butler H, Cherry JM, et al. Gene ontology: Tool for the unification of biology. Nature genetics. 2000;25(1):25.

8. The Gene Ontology Consortium. Expansion of the gene ontology knowledgebase and resources. Nucleic Acids Research [Internet]. 2017;45(D1):D331–8. Available from: http://dx.doi.org/10.1093/nar/gkw1108

9. Linsley PS, Speake C, Whalen E, Chaussabel D. Copy number loss of the interferon gene cluster in melanomas is linked to reduced t cell infiltrate and poor patient prognosis. PloS one. 2014;9(10):e109760.

10. Abbas AR, Baldwin D, Ma Y, Ouyang W, Gurney A, Martin F, et al. Immune response in silico (iris): Immune-specific genes identified from a compendium of microarray expression data. Genes and immunity. 2005;6(4):319.

11. Allantaz F, Cheng DT, Bergauer T, Ravindran P, Rossier MF, Ebeling M, et al. Expression profiling of human immune cell subsets identifies miRNA-mRNA regulatory relationships correlated with cell type specific expression. PloS one. 2012;7(1):e29979.

12. Mesko B, Poliska S, Váncsa A, Szekanecz Z, Palatka K, Hollo Z, et al. Peripheral blood derived gene panels predict response to infliximab in rheumatoid arthritis and crohn's disease. Genome medicine. 2013;5(6):59.

13. Julià A, Erra A, Palacio C, Tomas C, Sans X, Barceló P, et al. An eight-gene blood expression profile predicts the response to infliximab in rheumatoid arthritis. PLoS One. 2009;4(10):e7556.

14. Bienkowska JR, Dalgin GS, Batliwalla F, Allaire N, Roubenoff R, Gregersen PK, et al. Convergent random forest predictor: Methodology for predicting drug response from genome-scale data applied to anti-tnf response. Genomics. 2009;94(6):423–32.

15. Toonen EJ, Gilissen C, Franke B, Kievit W, Eijsbouts AM, Broeder AA den, et al. Validation study of existing gene expression signatures for anti-tnf treatment in patients with rheumatoid arthritis. PloS one. 2012;7(3):e33199.

16. MacIsaac KD, Baumgartner R, Kang J, Loboda A, Peterfy C, DiCarlo J, et al. Pre-treatment whole blood gene expression is associated with 14-week response assessed by dynamic contrast enhanced magnetic resonance imaging in infliximab-treated rheumatoid arthritis patients. PLoS One. 2014;9(12):e113937.
